# Origin and diversification of free-living stick spiders of Sri Lanka including the description of four new species of *Rhomphaea* L. Koch, 1872 and two new species of *Neospintharus* Exline, 1950

**DOI:** 10.1371/journal.pone.0273105

**Published:** 2022-09-07

**Authors:** Mathura Tharmarajan, Suresh P. Benjamin

**Affiliations:** National Institute of Fundamental Studies, Kandy, Sri Lanka; Nanjing Agricultural University, CHINA

## Abstract

Sri Lanka is a biologically diverse South Asian island, and together with the Western Ghats (Southern India) is one of the 36 world’s most biologically diverse areas. Here, we investigated the origin and diversification of *Rhomphaea* and *Neospintharus* of Sri Lanka using sequences of three genes: mitochondrial cytochrome c oxidase I (*COI*) and *16S* rRNA (*16S*); and nuclear *28S* rRNA (*28S*). Our phylogeny included 32 taxa (30 ingroup and 2 outgroup). We used Bayesian Inference and maximum likelihood methods to reconstruct the placement of species, divergence time estimations and their foraging behavior with an emphasis on species from Sri Lanka. Our phylogenetic hypothesis support the monophyly of Argyrodinae as well as the monophyly of *Rhomphaea*, where *Rhomphaea* is a sister group of *Neospintharus*. Further, our analysis also suggests that Sri Lanka was colonized by Argyrodinae several times. Additionally, the following new species are described: *Rhomphaea shanthi* **sp. nov.**, *Rhomphaea jacko* **sp. nov.**, *Rhomphaea martini* **sp. nov.**, *Rhomphaea marani* **sp. nov.**, *Neospintharus kandelensis* **sp. nov.** and *Neospintharus ohiyiaensis* **sp. nov.**

## Introduction

Sri Lanka is a biologically diverse South Asian island. Sri Lanka together with the Western Ghats (Southern India) is one of the 36 world’s most biologically diverse hotspots [1,2]. Large continental islands like Sri Lanka with faunas derived from a mixer of recent colonization by mainland species and long term *in situ* diversification have not been well studied [3,4]. However, such studies might offer new perspectives on processes generating island biodiversity [3,4]. Further, short-range endemics with very restricted distributions, as often found in Sri Lanka, may prove to be important flagship taxa for monitoring the effects of climate change and other threats on forest habitats [5].

Theridiidae Sundevall, 1833 commonly known as comb-footed spiders, is one of the largest families within Araneae comprising 2536 species classified in 125 genera [6]. Theridiids are small to medium sized spiders, about 1-15mm in total body length, most being less than 6 mm [7]. They are an extremely morphologically diverse [7,8] clade that originated in the Cretaceous [9] period and diversified during the Paleogene to modern times [7–10].

Spiders of the Theridiid subfamily Argyrodinae [9,11], consisting of the genera: *Argyrodes* Simon, 1864, *Ariamnes* Thorell, 1869, *Faiditus* Keyserling, 1884, *Neospintharus* Exline, 1950, *Rhomphaea* L. Koch, 1872 and *Spheropistha* Yaginuma, 1957, are known for their associations with other web-building spiders [11–13]. Species of these six genera are distinct, differing considerably in morphology and behavior. Members of the genera *Argyrodes* and *Faiditus* live in groups and are kleptoparasitic [14], whereas the genera *Rhomphaea*, *Ariamnes* and *Neospintharus* are solitary, free living and directly feed on spiders or other preys [15]. The behavior of the two species in the monotypic genera *Argyrodella* and *Deelemanella* are unknown [6,16,17]; they are not further treated here.

The study of stick spiders and their relatives has a long history. Exline and Levi [15] revised the genera *Argyrodes*, *Ariamnes* and *Rhomphaea*. They formally transferred all Argyrodinae genera in the genus *Argyrodes*, while recognizing species groups within it. However, their study was based mostly on New World material. Thereafter, Yoshida [16] retained the genus *Argyrodes* and resurrected *Ariamnes*, *Rhomphaea* and *Spheropistha*. Agnarsson [9] combined the *Cancellatus* and *Cordillera* species groups of Exline and Levi [15] to a single genus, *Faiditus*, and elevated the *Trigonum* species group to the genus *Neospintharus*. Further, Agnarsson’s [9] morphological study found some support to Yoshida’s [16] generic classification of Argyrodinae. However, recognising the genera of the subfamily Argyrodinae inclusive of species from the Oriental region is not easy.

Genus *Rhomphaea* was established by L. Koch in 1872 and currently contains thirty-three species distributed world-wide [6]. *Rhomphaea* differs from other Argyrodinae species by the following synapomorphies: elongated tibia, boomerang-shaped abdomen and rhomboid shape of egg sac [9]. *Rhomphaea* uses araneophagy and occasionally kleptoparasitism to feed [18]. Genus *Neospintharus* was established by Exline in 1950 and currently containing 13 described species recorded worldwide except for Africa and Australasia [6]. It differs from *Spintharus* by the relative size, position of eyes and the height of carapace which is much higher than other genera of subfamily Argyrodinae [9]. *Neospintharus* use araneophagy as their main foraging strategy [18].

We contend that our material, could be either placed in *Ariamnes*, *Rhomphaea* or *Neospintharus*. Unambiguous, placement in either of these three genera was probably hampered by the fact that previous definitions of all three genera was mostly based on New World material. Although, previous studies, including Su & Smith [18] included species from several zoogeographic regions, species from the Oriental region were under-represented. Further, no species from the Indian sub-region (South Asia) were included.

Therefore, the aim of this study was to undertake an integrative taxonomic review of the free-living stick spiders of Sri Lanka based on interpretations of morphological and molecular data. Further, we aim to infer the phylogenetic position of the newly discovered species using a multilocus molecular phylogenetic hypothesis. We also describe four new species of *Rhomphaea* and two new species of *Neospintharus*, recording both genera for the first time in Sri Lanka.

## Materials and methods

### Taxon selection

A multilocus molecular approach was used for this study. Target loci were selected based on prior molecular phylogenetic studies of Argyrodinae [18,19]. Fieldwork was conducted in all climatic regions of Sri Lanka. Specimens were collected by hand or by beating vegetation up to around two meters. The collected spiders were preserved in 70% or 100% ethanol for morphological or molecular downstream application. Taxon sampling consist of 32 terminal taxa comprising of 30 ingroup taxa (14 were newly sequenced for this study) of the subfamily Argyrodinae and two outgroup taxa (Table 1). We included 14 new terminals: 7 specimens of *R*. *shanthi* sp. nov., 2 specimens each of *R*. *marani* sp. nov. and *N*. *kandelensis* sp. nov., 1 specimen each of *R*. *jacko* sp. nov. and *R*. *martini* sp. nov. Additional sequences were obtained from [18,19] to represent a more geographically evenly distributed sample. Accession numbers for all sequences as well as locality information are given in Table 1.

**Table 1:** Details of exemplars used in this study including collection localities, GenBank accession numbers and National Institute of Fundamental Studies (NIFS) voucher numbers. Accession numbers in bold denote sequences generated for this study. All species belong to the family Theridiidae.

Species	Geographic origin	Voucher No	CO1	16S	28S
*Argyrodes rainbowi*	Philippines		KJ648430	-	-
*Argyrodes lanyuensis*	Taiwan	A1	KJ648424	KJ648322	KJ648356
*Argyrodes fasciatus*	Singapore		KJ648420	KJ648320	KJ648352
*Argyrodes* sp7	Thailand		KJ648435	KJ648332	KJ648362
*Argyrodes tripunctatus*	Philippines		KJ648436	-	-
*Ariamnes attenuatus*	Guyana		AY231033	AY231078	AY230946
*Ariamnes cylindrogaster*	Taiwan		KJ648437	KJ648365	KJ648365
*Ariamnes cylindrogaster*	Korea		JN817105	JN816902	JN816471
*Neospintharus syriacus*	Israel		KJ648442	KJ648371	-
*Neospintharus trigonum*	USA		AY231048	AY231077	AY230945
*Neospintharus trigonum*	USA		KJ648443	-	KJ648340
*Neospintharus kandelensis* sp. nov.	Sri Lanka, Kande Ela	IFS_THE_748		**MW045790**	**MW045771**
*Neospintharus kandelensis* sp. nov.	Sri Lanka, Kande Ela	IFS_THE_749		**MW045791**	**MW045772**
*Rhomphaea metalissima*	Guyana		AY231052	AY231083	AY230950
*Rhomphaea* sp.	Philippines		KJ648446	KJ648375	KJ648344
*Rhomphaea sinica*	Taiwan		KJ648445	KJ648374	KJ648343
*Rhomphaea sagana*	Taiwan			KJ648373	KJ648342
*Rhomphaea fictilium*	USA		KJ648341	KJ648372	KJ648408
*Rhomphaea jacko* sp. nov.	Sri Lanka, Ethagala	IFS_THE_029	**MW074333**	**MW477446**	**MW055432**
*Rhomphaea marani* sp. nov.	Sri Lanka, Nilgala forest	IFS_THE_024	-	**MW477445**	
*Rhomphaea marani* sp. nov.	Sri Lanka, Sinharaja	IFS_THE_292	**MW074329**	**MW477451**	
*Rhomphaea* sp.	Sri Lanka, Hiniduma	IFS_THE_769	**MW045780**		
*Rhomphaea martini* sp. nov.	Sri Lanka, Sita Eliya	IFS_THE_757	**MW045774**		**MW045793**
*Rhomphaea shanthi* sp. nov.	Sri Lanka, Kanneliya	IFS_THE_808	**MW058050**		
*Rhomphaea shanthi* sp. nov.	Sri Lanka, Kanneliya	IFS_THE_803	**MW058048**		
*Rhomphaea shanthi* sp. nov.	Sri Lanka, Sinharaja	IFS_THE_255	**MW074332**	**MW477449**	
*Rhomphaea shanthi* sp. nov.	Sri Lanka, Kanneliya	IFS_THE_802	**MW058047**		
*Rhomphaea shanthi* sp. nov.	Sri Lanka, Kanneliya	IFS_THE_800	**MW058046**		
*Rhomphaea shanthi* sp. nov.	Sri Lanka, Nilgala forest	IFS_THE_03	**MW074334**	**MW477443**	
*Rhomphaea shanthi* sp. nov.	Sri Lanka, Sinharaja	IFS_THE_236		**MW477447**	
**Outgroups**					
*Enoplognatha caricis*	Japan		AY231040	AY231096	AY230962
*Lactrodectus mactans*	USA		AY231046	AY231100	AY230966

### Morphology

Specimen preserved in 70% alcohol were examined using a Leica S9E binocular stereomicroscope (Leica Microsystems Limited, Wetzlar, Germany). Male palps (left) were dissected and immersed in Kaiser’s glycerol gelatin (Merck KGaA, Darmstadt, Germany), slide mounted, observed and illustrated with the aid of Leica DM3000 LED stereo microscope with an attached drawing tube. Highly sclerotized or darker areas of palps and epigynum were shaded with an HB pencil. The female epigastric region was dissected and digested in a pancreatin solution for about 3–7 days, slide-mounted and illustrated as described above. Digital images of the specimens were taken with a Leica MC170 HD camera mounted on a Leica M205C stereomicroscope using the software package Leica application suite, LAS version 4.6.2. Acquired image stacks of different depths (20–50 images per stack) were assembled using Helicon Focus (version 6, Helicon Soft Ltd) to create a single image with the entire specimen in focus. Final images were edited using Adobe Photoshop Version CS6. Body length was considered as total length of prosoma + total length of opisthosoma (excluding spinnerets). In case of *Rhomphaea*, as their opisthosoma is elevated and folded, opisthosoma length was measured in two parts, anterior opisthosoma length (range from the point of pedicel in opisthosoma–spinnerets) and posterior opisthosoma length (point of spinnerets–posterior opisthosoma tip). Leg measurements are given in the following order: total (femur, patella, tibia, metatarsus, tarsus).

Descriptions of morphological terminology follows Agnarsson [9]. All measurements are in millimetres. Types and other specimens of the new species described herein are currently in the NIFS and will be deposited in the National Museum of Sri Lanka, Colombo.

### Molecules

Partial fragments of the *28S* ribosomal RNA (*28S*) and mitochondrial protein-coding gene cytochrome *c* oxidase subunit 1 (*COI*) and *16S* ribosomal RNA (*16S*) were amplified. *COI* and *16S* gene regions are more suitable to resolve more recent evolutionary events, whereas *28S* is more effective in resolving deeper nodes in phylogenetic trees [20]. Details of each primer pair used, expected amplicon length (bp, number of base pairs) annealing temperature/time, primer sequences and related references are given in Table 2.

**Table 2:** Gene targets, PCR conditions and primer data used in this study.

Gene	Primer name	Primer sequence	Annealing Temperature (°C)	Reference
*CO1*	LCO1-1490 HCO- 2198	GGTCAACAAATCATAAAGATA TAAACTTCTGGATGTCCAAAGAATCA	50–60	[22] [26]
	LCO1- 1490 HCO-2776	GGTCAACAAATCATAAAGATA GGATAATCAGAATATCGTCGAGG	57	[22] [27]
	LCO1-1490 HCO1-out out	GGTCAACAAATCATAAAGATA GTAAATATATGRTGDGCTC	46	[22] [28]
*16S*	NIJ-12581 LRN-12945R	CCTTTAACGAATTTGAATATA CGACCTCGATGTTGAATTAA	46	[27]
	16Sar 16Sb	CGCCTGTTTATCAAAAACAT CTCCGGTTTGAACTCAGATCA	48	[28] [28]
*28S*	28Sc 28So	GGTTCG ATT AGT CTT TCG CC GAAACTGCTCAAAGGTAAACGG	46	[21,29] [21,29]
	28Sa 28Sr7bi	GACCCGTCTTGAAACACGGA GACTTCCCTTACCTACAT	54	[21] [21]
	28So 28Sr7bi	GAAACTGCTCAAAGGTAAACGG GACTTCCCTTACCTACAT	54	[21]

Genomic DNA was extracted from 100% ethanol-preserved leg tissue using DNeasy^®^ Blood and Tissue Kit (Qiagen, Hilden, Germany). Polymerase chain reaction (PCR) was carried out using primers used in previous studies [20–23]. All sequences were edited and aligned using Genious 11.1.5 and Mesquite v 3.51 [24]. The protein coding *CO1* sequences were aligned easily. *16S* and 2*8S* sequences were subsequently treated with Gblock 0.91b [25] to cull ambiguous positions. Gblock parameters were defined as follows: minimum number of sequences for a conserved position (50%), maximum number of contiguous non-conserved positions (10), minimum number of sequences for a flanking position (46), minimum length of a block (2), allowed gap positions (With Half) and similarity matrices were used. All sequences have been deposited in Gene Bank and their accession numbers are given in Table 1.

### Phylogenetic analysis

Bayesian and Likelihood methods were used for single and concatenated gene matrices. Prior to likelihood and Bayesian analyses, Partition finder software v 2.1.1 [30] was run to find the best fit model for each partition using linked Bayesian Information Criterion (BIC) (Table 3). We tested the codon position specific models for each gene at 1^st^, 2^nd^, 3^rd^ positions. The evolutionary models for each codon positions were then applied in the subsequent Bayesian and Maximum likelihood analysis. The results of model selection and priors used for partitioned are listed in the Table 3.

**Table 3:** Best schemes and models of gene partitions from partition finder.

Analysis	Partition and length (bp)	Fragment Length (base-pair)	Model Chosen by Partition finder model test	Model implemented in Mr.Bayes and IQtree
**MrBayes/** **IQtree**	*CO1* gene: 1^st^ codon	1069	F81+G	GTR+G
	2^nd^ codon		K81uf +G	
	3^rd^ codon		TIM+G	
	*16S*	536	GTR+I+G	GTR+I+G
	*28S*	544	GTR+I+G	GTR+I+G

Phylogenetic trees were inferred using Bayesian method in MrBayes v 3.2 [31] and Maximum likelihood method in IQtree v 1.6.12 [32]. We conducted these analyses for each gene separately and also for the concatenated data matrix. Each Bayesian analysis comprised of two independent Markov chain Monte Carlo (MCMC) chains and 1×10^6^ generations per run. In all MrBayes analyses we discarded the first 25% of the sampled trees as burn in. We sampled a tree every 1×10^3^ generations in each analysis, then visually inspected the likelihood scores and posterior probability scores of trees in Fig Tree v 1.4.2 [33]. Maximum likelihood analysis for each dataset were conducted in IQtree with 1000 bootstrap replications.

### Divergent time estimation

Estimates of divergence times were computed on the concatenated data matrix using BEAST v. 1.8.2. Beauti v. 1.8.2 [34] was used to generate the XML file. The clock models and substitution rate were unlinked and substitution models for each gene were set up as in MrBayes. The fossil information from a previous study was used to calibrate the tree [10,18]. The oldest date at the node representing the lineage of members of genera *Rhomphaea* and *Neospintharus* was set at 12.5 Mya with normal distribution prior and an arbitrary standard deviation of 0.01. We performed two independent runs of the analysis and in each run the first 25% of the trees were removed as burn-in. The MCMC chain length of each run was 1×10^7^ with the frequency of 1×10^3^. The generated tree files were then used to generate a maximum clade credibility tree using TreeAnnotator v. 1.8.2 [34]. The final time-scaled tree was visualized using Figtree.

### Distribution of species

The occurrence of the sampled species in Sri Lanka was mapped in QGIS v 3.14.16 [35] using the coordinates of sampled localities obtained from field works. Localities with uncertain coordinates were resorted to approximation of the points using Google Earth [36].

The list of abbreviations used in the study is given in Table 4.

**Table 4:** Table of abbreviations used in the manuscript.

Abbreviation	Description
**DFC**	Department of Forest Conservation
**DWLC**	Department of Wild Life Conservation
**FR**	Forest Reserve
**NIFS**	National Institute of Fundamental Studies
**NMSL**	National Museum of Sri Lanka
**AME**	anterior median eyes
**ALE**	anterior lateral eyes
**BC**	bulb-cymbium
**C**	conductor
**CD**	copulatory duct
**CO**	copulatory opening
**Cy**	cymbium
**Chd**	cymbial hood
**Chk**	cymbial hook
**E**	embolus
**EA**	embolic apophysis
**ETA**	extra tegular apophysis
**FD**	fertilization duct
**MA**	median apophysis
**PLE**	posterior lateral eyes
**PME**	posterior median eyes;
**S**	spermathecae
**SD**	sperm duct
**SC**	sub conductor
**ST**	sub tegulum
**T**	tegulum
**Tb**	trichobothria
**THE**	Theridiidae
**Tp**	tegular pit
**Ta**	tarsus
**Ti**	tibia
**TTA**	theridiid tegular apophysis

### Nomenclatural acts

The electronic edition of this article conforms to the requirements of the amended International Code of Zoological Nomenclature, and hence the new names contained herein are available under that Code from the electronic edition of this article. This published work and the nomenclatural acts it contains have been registered in ZooBank, the online registration system for the ICZN. The ZooBank LSIDs (Life Science Identifiers) can be resolved and the associated information viewed through any standard web browser by appending the LSID to the prefix "http://zoobank.org/". The LSID for this publication is: urn:lsid:zoobank.org:pub: urn:lsid:zoobank.org:pub:4399F1E7-0B98-4A9A-B098-ECA7FA18E6BB. The electronic edition of this work was published in a journal with an ISSN, and has been archived and is available from the following digital repositories: PubMed Central, LOCKSS [author to insert any additional repositories].

## Results

### Phylogenetic analysis

The assembled matrix of the concatenated mitochondrial and nuclear markers included 69 sequences of 22 taxa; 22 of these sequences were newly generated for this study. The lengths of targeted fragments after excluding primers and Gblock treatment were as follows: *16S* ~536bp, *28S* ~544bp and *CO1* ~1067bp. The assembled matrix of mitochondrial and nuclear markers includes 32 taxa (30 ingroups and 2 outgroups). The total length of final matrix was 2147bp. The best-fit model for all data matrixes are given in Table 3.

**Fig 1:**
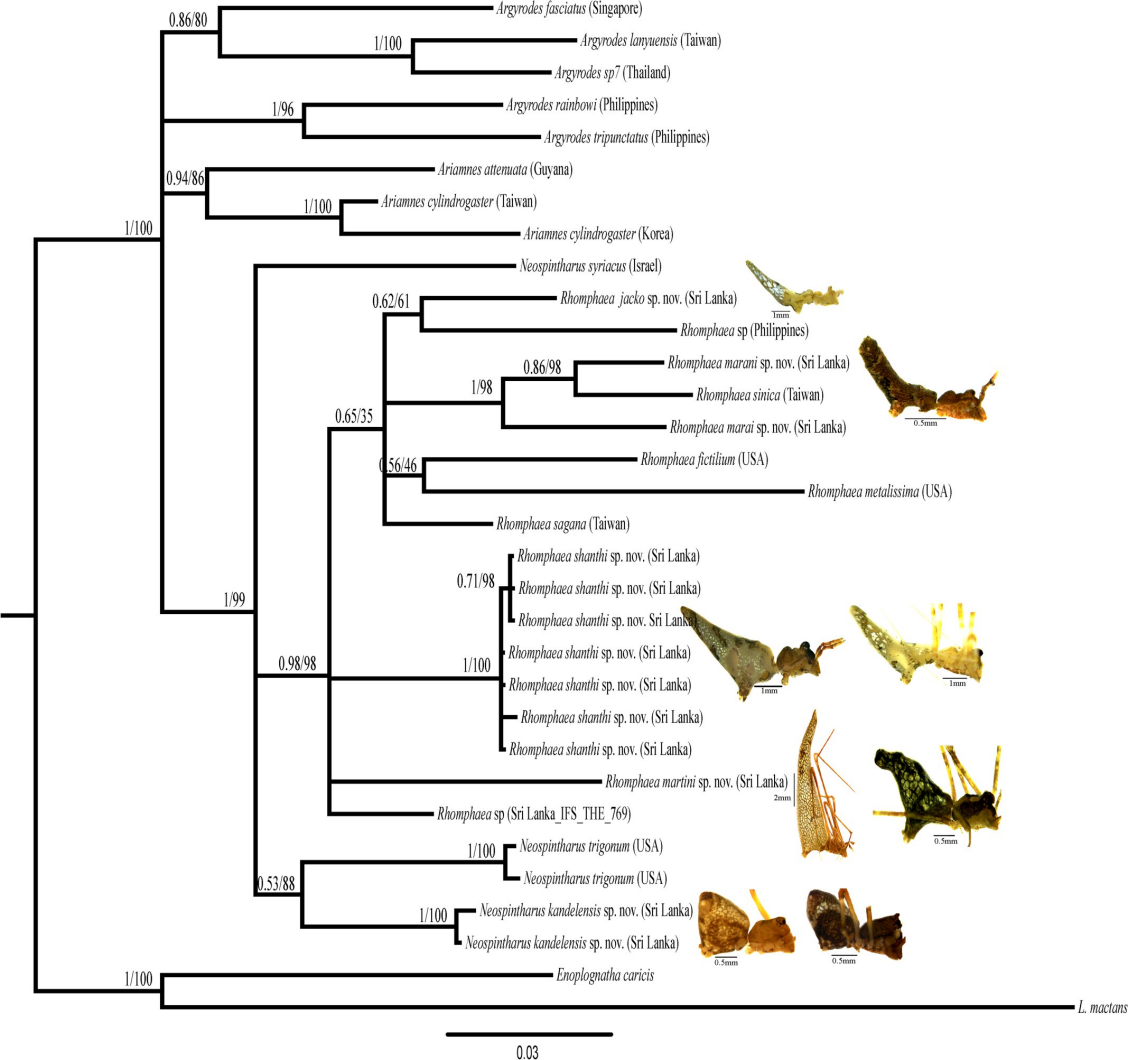
The phylogenetic tree inferred by Bayesian analysis of the combined molecular data (*COI*, *16S* and *28S*) in MrBayes. The numbers above nodes are posterior probability values / bootstrap values.

**Fig 2:**
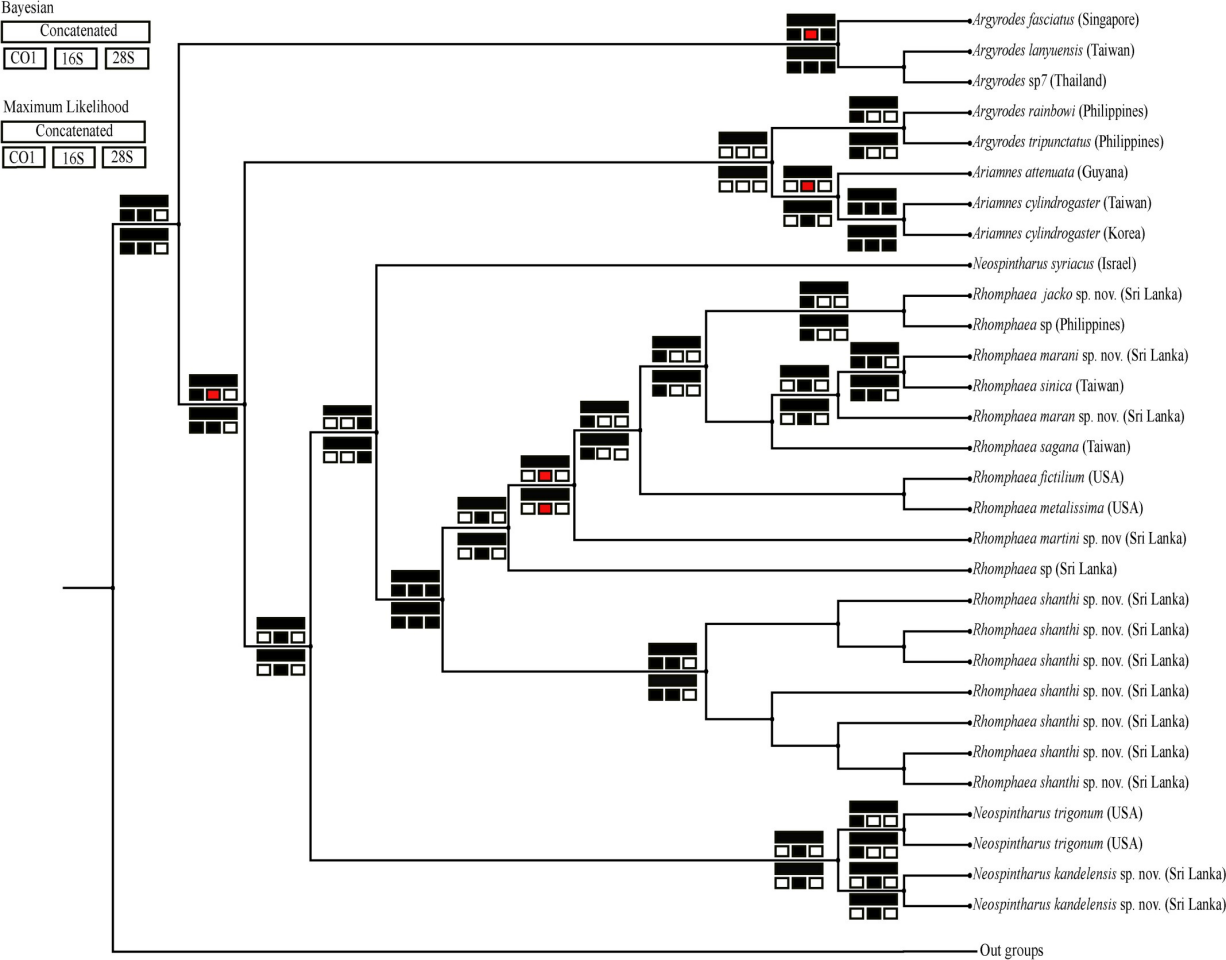
Summary phylogenetic tree of the Bayesian and Maximum Likelihood analyses of the three individual genes as well as the concatenated molecular data. The tree recovered from the ML analysis of the concatenated molecular data is used here as it is better resolved. Key: “Navajo rugs” indicate posterior probability support of a node is larger than 0.7 in Bayesian analysis and larger than 70 in ML analysis of the individual gene or in concatenated data (black), or nodal supports values between 0.6–0.7 in Bayesian and 60–70 in ML analysis (red), absence of a given node in the tree specified in the legend (white).

The phylogenetic tree resulting from the Bayesian analysis of the combined data matrix is presented in Fig 1. The phylogenetic tree resulting from the ML analysis is presented in S1 Fig. Topologies of all trees are summarized in Fig 2. The trees resulting from both analyses are generally concordant with each other as well as with previous analyses [18,19] and are well-supported, with respect to our taxa of interest. Thus, the topology of both the Bayesian and ML trees are discussed together; the differences are presented in the summary tree (Fig 2). Both trees resulting from the analysis of the concatenated data recover the monophyly of *Rhomphaea* as well as *Ariamnes* post inclusion of Sri Lankan taxa. (Bayesian posterior probability = 0.98 and ML bootstrap support = 98; Figs 1 and S1). Sri Lankan *Rhomphaea* included in this study do not form a single clade, instead popup at various points within the *Rhomphaea* clade.

However, monophyly of *Neospintharus* and *Argyrodes* are not recovered. The Sri Lanka species of *Neospintharus* included in the analysis, *N*. *kandelensis* sp. nov. groups with *N*. *trigonum*, excluding *N*. *syriacus* (bootstrap value of ML = 88; posterior probability = 0.53; Figs 1, S1 and S7).

### Divergent time estimation

**Fig 3:**
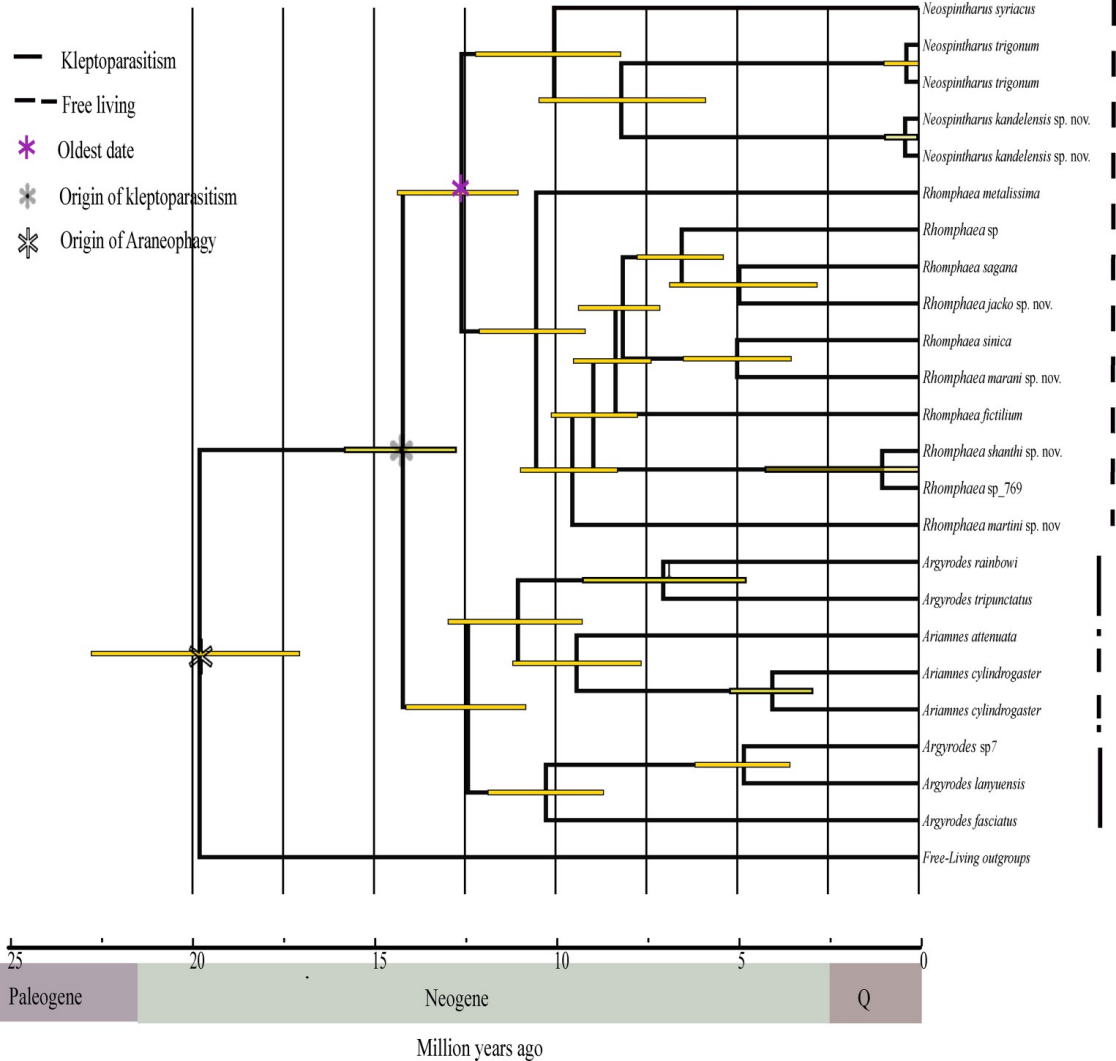
A divergent time calibrated phylogeny estimated in BEAST. Times of origination using the age of the oldest date extracted from a previous study [20]. Placement of oldest date marked as purple asterisk on the node. The yellow bar on the node shows the 95% highest probability density interval of the estimated time.

Results from our dating analysis are presented in Fig 3. The dated phylogeny suggests that origin of Argyrodinae occurred around 19.6 Mya (96% Highest Probability Density, HPD interval of 22.5–16.75 Mya) which was between the late Paleogene and early Neogene (Fig 3). The origin of kleptoparasitism was dated in between the early to late Neogene (mean = 14.09 Mya with 95% HPD interval of 15.75–12.75 Mya). Based on our dating analysis, araneophagy may have evolved at least 5 My earlier than kleptoparasitism. Further, the origin and diversification of the genus *Rhomphaea* in Sri Lanka was in the early Neogene at around 10–5 million years ago.

## Discussion

### Phylogenetic analysis

Phylogenetic relationships inferred in this study largely confirm previous findings of Su & Smith [18]. However, the phylogenetic placement of *Rhomphaea* and *Neospintharus* yielded mixed results. After the addition of 12 new terminals, comprising of sequences for five species to the matrix of Su & Smith [18], the resulting trees for both analyses recover a fairly well supported *Rhomphaea* clade. All putative Sri Lankan species cluster within this clade (Fig 1).

Our results provides further evidence in the classification of argyrodines by recognizing four distinctive generic clades of *Argyrodes*, *Ariamnes*, *Rhomphaea* and *Neospintharus*. However, our results indicate that circumscription of genera within Argyrodinae needs further attention. This is also suggested by the polytomies present in our preferred phylogenetic hypothesis. We found that, topology and clade composition of our phylogeny differs somewhat from previous studies [9,18]. However, we have here included only a limited sample of the diversity of Argyrodinae, focusing on taxa closely related to the stick spiders of Sri Lanka.

Members of genera *Ariamnes* and *Rhomphaea* are solitary, free living and specialized in ‘silk throwing’ [37,38] behavior associated with araneophagy and kleptoparasitic *Argyrodes* species [18,37,39]. *Neospintharus* shows both traits; directly feeding on prey and occasionally ‘silk throwing’ [18,37]. Unfortunately, behavioral data for Sri Lanka species included here are still not available.

This study as well as unpublished data on the kleptoparatic *Argyrodes*, represents the most comprehensive molecular survey conducted for the subfamily Argyrodinae of Sri Lanka. Our molecular genetic data will enable further evolutionary studies on the diversification of the subfamily. The discovered molecular and morphological diversity, new species and their restricted range distributions with species inhabiting low land and highland cloud forest call for a larger assessment of their evolutionary history and conservation, based on broader sampling regimes and genomic analyses. Further, future studies will also have to address the systematics of the Argyrodinae as no clear evidence supporting the monophyly of generic groups or species groups were found.

### Divergent time estimation

Based on our calibration (Fig 3) Sri Lankan *Rhomphaea* and *Neospintharus* species appear to have diverged very recently (less than 2Mya). Further, although our results are preliminary, it appears that both genera colonized Sri Lanka independently. Our study suggests that the new species of *Rhomphaea* and *Neospintharus* in Sri Lanka may have originated in the island; however further study needed to test this hypothesis.

## Taxonomy

### Theridiidae Sundevall, 1833

#### Subfamily Argyrodinae Simon 1894; Yoshida 2001

*Genus Rhomphaea L*. *Koch*, *1872*. **Diagnosis.** *Rhomphaea* species differ from other Argyrodinae by the following characters: prosoma of males with elevated or projected eye region or sometimes with a lobe-like structure; clypeus slanted anteriorly; opisthosoma boomerang shaped [8], elongated, triangular or cylindrical, posterior half of opisthosoma (extending beyond the spinnerets) at least three times longer than the anterior half, sometimes tapered to form a single tip with a seta, in most of the species orientation of anterior and posterior opisthosoma halves form angle at spinnerets; elongated palpal tibia, embolus spiral directed clockwise in left palp, membranous conductor, in some species apex of TTA enlarged; epigynum with a socket-like ventral membranous structure (scape); rhomboid shaped egg sac [8,9].

**Description.** Medium to large **s**piders. Prosoma pale yellow, with lateral black bands arising from the back of eye band. Eye band projected anteriorly, fused laterals. Clypeus slanted, projected anteriorly, distinct bi-lateral blackish-brown bands. Sternum slightly convex, blackish brown dusty flecks. Labium fused with sternum. Opisthosoma similar to prosoma in coloration with silver and brown-black flecks, triangular or cylindrical shaped, anterior and posterior opisthosoma divided by spinnerets, posterior half 3–5 times longer than anterior. Legs pale-yellow, reddish brown bands/patches, leg formula I, IV, II and III. Pedipalp and legs similar in color and banding pattern. Palpal tibia elongated, narrow, tapered towards patella. Tibial rim scoop-shaped or flat with 3–5 retro lateral trichobothria. Cymbium entire with a characteristic cymbial hook or hood. Paracymbium absent. Membranous conductor closely associated with embolus tip. Embolusbifurcated in some species (embolus apophysis [9]), base lobbed, embolus spiral tip elongated and needle-like. TTA membranous or slightly sclerotized. Female epigynum not well sclerotized, ventral plate with a socket-like membranous hood (scape) [40], inconspicuous copulatory openings inside it. A pair of spermathecae oval shaped and larger. Copulatory duct long and encircled or short and straight. Fertilization duct shorter and twisted or straight.

**Composition.** 33 species; see World Spider Catalog [11] for a listing of all species.

**Distribution.** Worldwide; See World Spider Catalog [11].

### *Rhomphaea shanthi* sp. nov.

**Fig 4:**
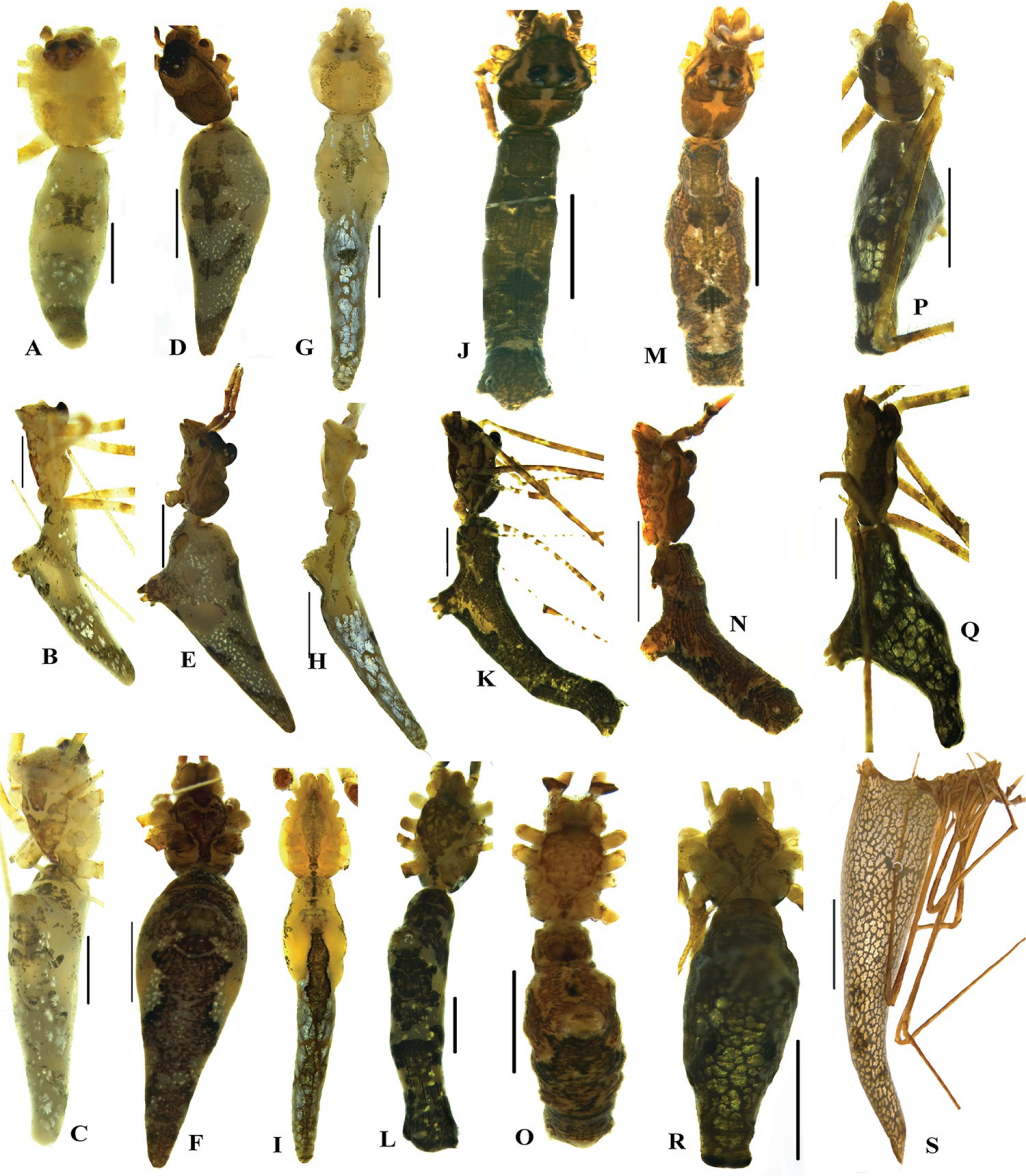
Habitus of *Rhomphaea* species of Sri Lanka. A–F *R*. *shanthi* sp. nov. G–I *R*. *jacko* sp. nov. J–O *R*. *marani* sp. nov. P–S *R*. *martini* sp. nov. A–C, G- I, J–L, P–R male habitus; D–F, M–O, S female habitus. A, D, G, J, M, P dorsal view; B, E, H, K, N, Q, S lateral view; C, F, I, L, O, R ventral view. Scale bars = 1 mm (D–F, B, G–I, J, L, M–O, P, R), 2 mm (S), 0.5 mm (A, C, N, Q).

**Fig 5:**
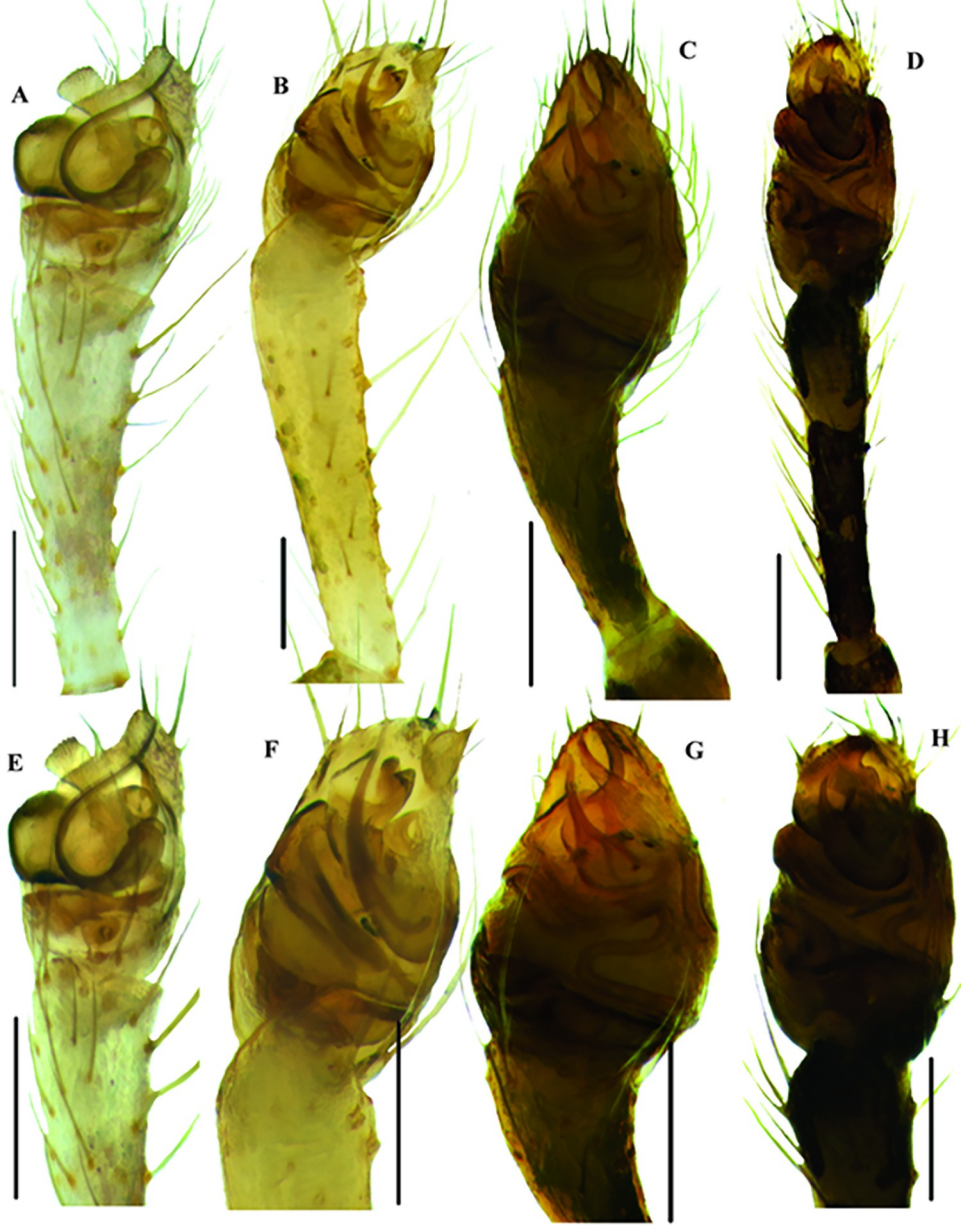
Male palpal morphology of *Rhomphaea* species of Sri Lanka. A–H male palps of *Rhomphaea* species, ventral view. A–B, *R*. *shanthi* sp. nov.; C–D, *R*. *jacko* sp. nov.; E–F, *R*. *martini* sp. nov., G–H, *R*. *marani* sp. nov. Scale bars = 0.2 mm (A–H).

**Fig 6:**
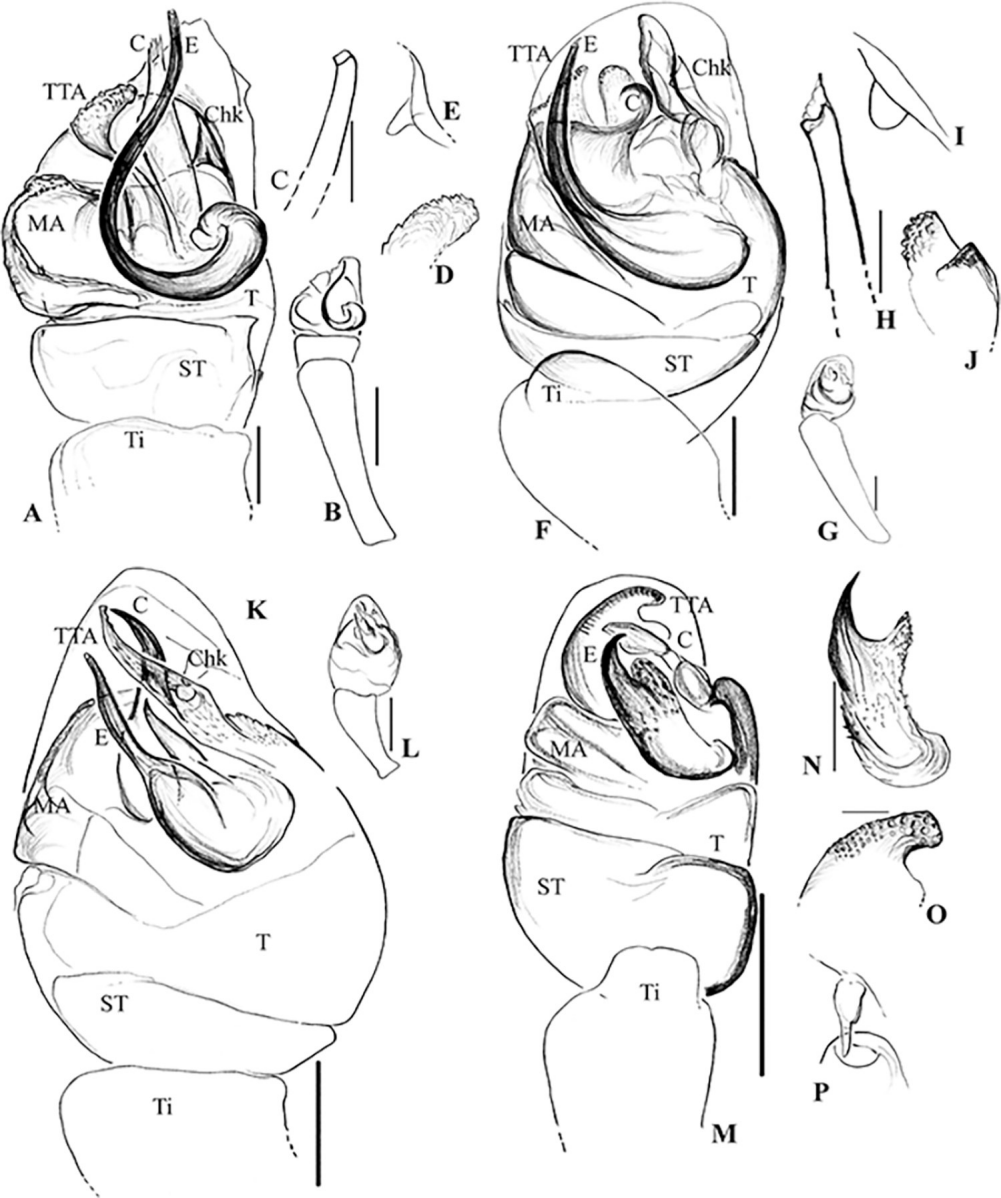
Male palpal morphology of *Rhomphaea* species in Sri Lanka. A–E, *R*. *shanthi* sp. nov.; A, left palp, ventral view; B, tibia; C, tip of embolus; D, tip of Conductor; E, cymbial hook. F–J, *R*. *jacko* sp. nov.; F, left palp, ventral view; G, tibia; H, tip of embolus; I, cymbial hook, J, tip of TTA; K–L, *R*. *martini* sp. nov.; K, left palp, ventral view; L, tibia. M–P, *R*. *marani* sp. nov.; N, embolus; O, tip of TTA; P, cymbial hook. Abbreviations = C, conductor; CD, copulatory duct; Chd, cymbial hood; Chk, cymbial hook; E, embolus; MA, median apophysis; ST, sub-tegulum; T, tegulum; Ti, tibia; TTA, theridiid tegular apophysis. Scale bars = 0.1 mm (A, F, K, M), 0.2 mm (B, G, L), 0.02 mm (C, E, H, I, J, N, O, P).

**Fig 7:**
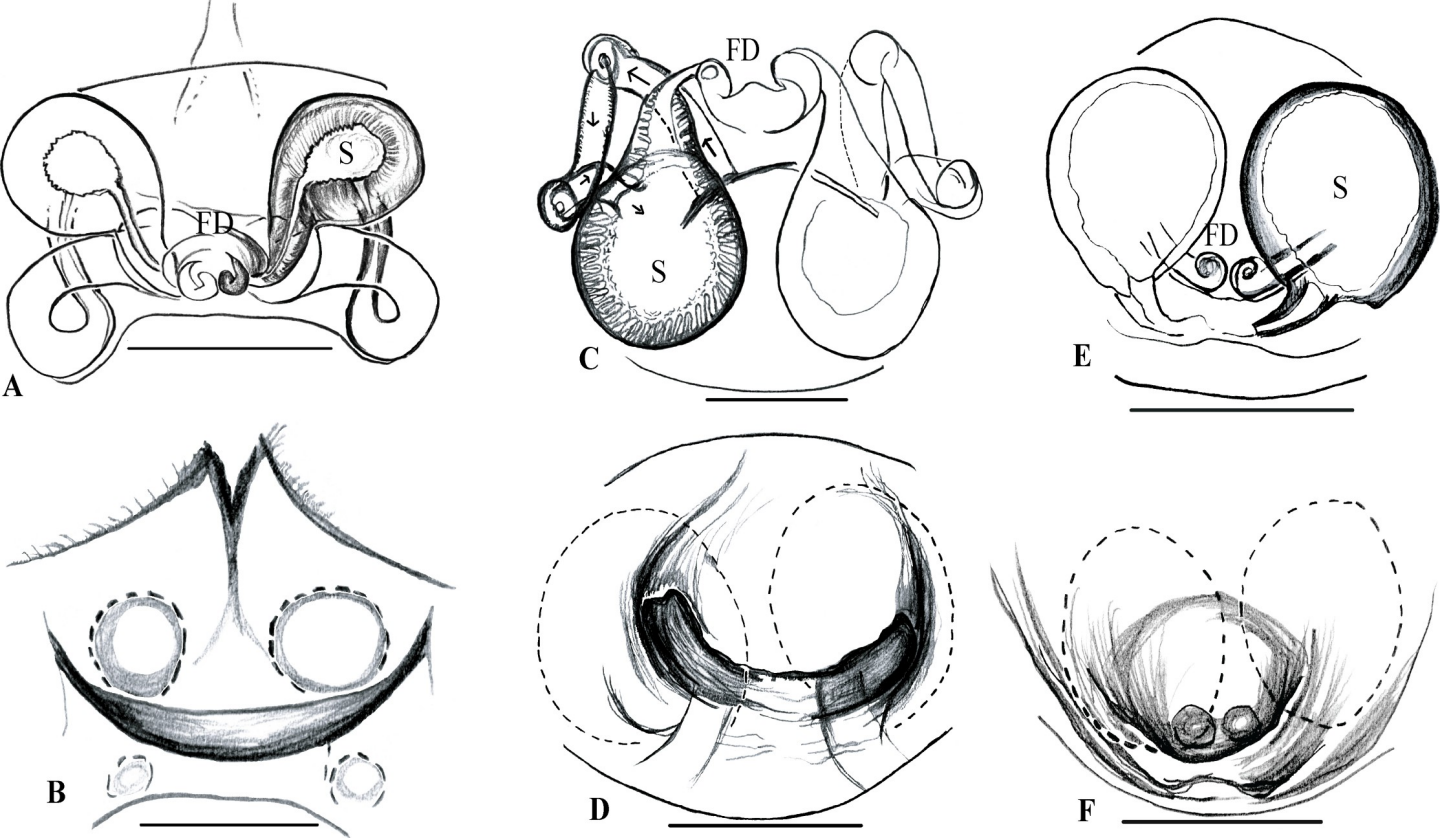
Female genital morphology of *Rhomphaea* species of Sri Lanka. A–B epigynum of *R*. *shanthi* sp. nov; A dorsal view; B ventral view. C–D epigynum of *R*. *martini* sp. nov.; C dorsal view, D ventral view. E–F epigynum of *R*. *marani* sp. nov.; E dorsal view; F ventral view. Abbreviations = S, spermathecae; FD, fertilization duct. Scale bars = 0.1 mm (A, C, F), 0.2 mm (B).

Figs 4A–4C, 4D–4F, 5A–5B, 6A–6E, 7A and 7B

LSID: urn:lsid:zoobank.org:act:DCB87226-E4B7-4ADB-8305-E1704DE08E53

**Type material. Holotype:** ♂ (IFS_THE_802): Sri Lanka, Southern Province, Galle District, Kanneliya FR, 06°15’04’’N, 80°20’18’’E, 30m, 18 February 2020, Hand collection, leg. S. P. Benjamin *et al*. **Paratype:** ♀ (IFS_THE_803): Same locality and collection data as in holotype. **Other material examined. SRI LANKA: *Uva province*:** 1♀ (IFS_THE_003), Monaragala District, Nilgala FR, 07°11’08.79’’N, 81°24’24’’E, 122m, 23 January 2018, S. P. Benjamin *et al*. ***Western Province*:** 1♂, 1♀ (IFS_THE_34–35), Colombo district, Labugama FR, 06°83’N, 80°18’E, 183m, 21 April 2016, beating, S. P. Benjamin & N. P. Athukorala. ***Sabaragamuwa Province*:** 1♀ (IFS_THE_46), Rathnapura District, Gilimale FR, 06°45’55.8’’N, 80°25’45.5’’E, 110m, 27 February 2007, hand collection, S. P. Benjamin & Z. Jaleel; 2♀ (IFS_THE_236, IFS_THE_255), Rathnapura District, Sinharaja FR, Kudawa, primary forest, 06°25’36’’N, 80°25’18’’E, 5 March 2019, beating, S. P. Benjamin *et al*; 1♀ (IFS_THE_268), Rathnapura District, Palabaddala, 22 August 2012. ♀ (IFS_THE_826), Rathnapura District, Eastern Sinharaja, Morning side Section, 06° 24’ 10.1412’’N, 80°37’41.1816’’E, 9 August 2021, beating, S. P. Benjamin et al. ***Southern Province*:** 2♀ (IFS_THE_800, IFS_THE_808), Sri Lanka, Galle District, Kanneliya FR, 06°15’04N, 80°20’18’’E, 30m, 18 February 2020, hand collection, S. P. Benjamin *et al*.

**Etymology.** This species is named after the first author’s mother, Shanthinimala Tharmarajan. Used as a noun in apposition.

**Diagnosis.** Males of *R*. *shanthi* sp. nov. differ from others congeners by comparatively larger, yellowish, triangular opisthosoma with reddish brown and silver flecks (Fig 4A–4C), embolus; longer, narrower and “inverted-question-mark-shaped”. TTA apex with 3–5 small narrow finger-like projections (Fig 6A–6E). Females differ from others congeners by the CD which is with one loop, enters S posteriorly (Fig 7A).

**Description.** Male (holotype): based on alcohol preserved specimens. Prosoma: length 0.9, width 0.7; smooth, longer than wide, similar to opisthosoma in coloration, continuation of distinct bilateral black stripes from clypeus, two humps separated by a horizontal transverse thoracic depression where posterior hump slightly higher. Clypeus slightly convex, moderately slanted, similar color of prosoma, bilateral longitudinal stripes. Labium rectangular, distally not swollen, rebordered, fused with sternum. Sternum reddish brown, longer than wide. Ocular area elevated, projected anteriorly beyond clypeus, medians larger than laterals, measurements: AME 0.1, ALE 0.04, PME 0.1, PLE 0.04, PME–PME 0.1, PLE–PLE 0.2, AME–AME 0.1, ALE–PME 0.1, ALE–PLE 0. Opisthosoma slightly cylindrical, boomerang shaped, extending beyond spinnerets, pale yellow with silver and blackish spots, anterior opisthosoma wider, tapered towards the end, posterior tip slightly blunt, opisthosoma length: pedicel–spinnerets 0.9, spinnerets–abdominal tip 1.8, opisthosoma width 0.6. Angle pedicel–spinnerets–posterior opisthosoma tip approximately about 110°–120°. Spinnerets in sub-apical region of opisthosoma. Legs pale yellow with reddish brown bands, patella brown. Leg measurements: leg I: missing in the material leg II: 5.8 (2.3, 0.3, 1.4, 1.2, Ta 0.6); leg III: 2.9 (1.2, 0.2, 0.5, 0.6, 0.4); leg IV: 8.2 (3.4, 0.4, 1.8, 1.8, 0.8). Palp stem similar to legs in band coloration. Palpal tibia long, distal end slightly wider than base, base narrower than the patella at articulation. Tibia rim uniform to slightly asymmetric and facing bulb, upper margin, with a regular row of 2–4 long strong setae, three retro lateral trichobothria. Cy entire, retromargin with the distal Chk facing downward inside the cymbium interacting in bulb cymbium lock mechanism, cymbial tip dorsal margin with 4–5 strong elongated setae. ST retrolateral margin slightly rounded and lobed. T less than half of cymbial cavity. C; membranous, tube-like, distal head scaly and enlarged, heavily ridged, not well sclerotized when compare to E and MA, located between E and MA, originates from tegular margin. MA unbranched, flexibly attached sclerite, mesial originated on T, outgrowth of T, small loop of SD can be seen, accommodates Chk corresponding pit. TTA membranous, vertical tube-like, unbranched, apex with 3–5 finger-like projections, resides back of E, attached to T, loosely associated. E rests in front of bulb, originates retroventrally, well sclerotized, longer than half of cymbium length, “inverted question mark” shaped, lobed base, spiral narrow cylindrical, tip hook-like. Palp measurements: length of Ti 0.5, length of Cy 0.3.

**Female (paratype).** based on alcohol preserved specimens. As in male except for the following: Prosoma brown, narrow transverse thoracic depressions, divided into 3 convex regions, length 1.4, width 0.7. Clypeus darker than prosoma, slanted. Sternum similar to ventral opisthosoma in coloration, longer than wide. Labium semicircular, fused with sternum. Pedipalp reddish brown shaded, length 1.5–2.0. Ocular area darkened, projected, measurements: AME 0.14, ALE 0.08, PME 0.08, PLE 0.08, PME–PME 0.14, PLE–PLE 0.32, ALE–PME 0.12, ALE–PLE 0. Opisthosoma pale yellow with small shiny silvery spots, sparsely arranged small setae, venter and posterior tip shaded in reddish-brown and small silvery spots, triangular, elevated, posterior two times longer than anterior and tapered up to tip, angle between pedicel–spinnerets–posterior tip approximately about 50°–60°, length; pedicel–spinnerets 1.9, spinnerets–posterior tip 4.4, width 0.6. Legs yellow shaded with reddish brown bands. Leg measurements: leg I: 16.8 (6.4, 0.4, 5.6, 2.9, 1.5), leg II: 9.3 (3.4, 0.5, 2.5, 1.9, 1.0), leg III: 4.2 (1.5, 0.3, 0.9, 1.0, 0.5), leg IV: 12.8 (5.2, 0.6, 3.2, 2.7, 1.1). Epigynum as in (Fig 7B), ventral plate smooth (Fig 7B). CD with one loop, enters S posteriorly (Fig 7A).

**Distribution.** See Fig 11.

### *Rhomphaea jacko* sp. nov.

urn:lsid:zoobank.org:act:605E8200-C599-4A1D-8D5D-5C93CB13F784

Figs 4G–4I, 5C, 5D and 6F–6J

**Type material. Holotype:** ♂ (IFS_THE_029), Sri Lanka, North Western Province, Kurunegala District, Kurunegala, Ethagala Mountains, 07°29’11.23’N, 80°22’21.64’’E, 190m, 03 June 2015, beating, N. P. Athukorala *et al*.

**Other material examined. Sri Lanka: *Central Province*:** 1♂ (IFS_THE_31), Matale District, Riverstone, Knuckles Range, 07°31’47.82’’N, 80°44’23.32’’E, 1088m, 23 March 2010, Hand Collection, P. M. H. Sandamali *et al*; 1♂ (IFS_THE_62), Matale District, Cardamom plantations, 3–4 September 2003, beating, S. P. Benjamin *et al*.

**Etymology.** This species is named after the first author’s pet dog Jacko (2015–2020). Used as a noun in apposition.

**Diagnosis.** Males of *R*. *jacko* sp. nov. closely resembles *R*. *sagana* but differ from other congeners by the length (longer) and shape of opisthosomsa (sickle-shaped, narrow, tapered and posterior part approximately about 8–10 times longer than anterior). The palpal sclerites (shape and location of embolus, conductor). Conductor (clearly visible, distal portion with hook-like apophysis (sub conductor). In *R*. *sagana*, conductor is not clearly visible [40]. Further, the lobed embolus base, embolus spiral narrow and grooved, the needle-like embolus tip is diagnostic.

**Description.** Male (holotype): based on alcohol preserved specimens. Prosoma yellow, flat but slightly convex in the middle with projected eye region, midline of dorsal prosoma and sternum with bilateral narrow faint-black lines, prosoma width equal to opisthosoma width, prosoma length 1.35, prosoma width 0.9. Clypeus slanted, length 0.26. Sternum narrow, 3 times longer than wide, widest at the 2^nd^ coxae and pointed between 4^th^ coxae. Labium semicircular and re-bordered. Eyes positioned on upper projection of prosoma, wider eye band, larger medians, measurements: AME 0.1, ALE 0.08, PME 0.08, PLE 0.08, PME–PME 0.1, PLE–PLE 0.3, ALE–PME 0.08, ALE–PLE 0. Opisthosoma sickle shaped, triangular, very long, posterior opisthosoma almost 10 times longer than anterior part, posterior opisthosoma covered with shiny larger silver spots, cylindrical, elongated upwardly and tapered towards the posterior tip with a characteristic long strong seta, length; pedicel–spinnerets 0.9, spinnerets-posterior opisthosoma tip 4.3, opisthosoma width 0.65. Legs; long, yellow, prominent light black bands (similar to the stripes on prosoma), Leg measurements: leg I: 10.45 (4.7, 0.35, 2.25, 2.1, 1.05), leg II: 6.34(2.3, 0.45, 1.7, 1.5, 0.8) leg III: 3.65 (1.45, 0.3, 0.7, 0.7, 0.5), leg IV: 6.83 (2.94, 0.3, 1.7, 1.44, 0.45). Palp: length of Ti 0.7, length of Cy 0.32, with light black bands. Tibia scoop shaped, distal end slightly wider than base, rim strongly asymmetric, protruding, strongly exaggerated on one side, characteristic row of long strong setae, 4–5 retro lateral trichobothria, two pro-lateral trichobothria. Cymbium entire, retromargin with a small distal cymbial hood. Chk faces downward inside Cy, distal portion blunt. T less than half of cymbial cavity. C located between E and MA, slightly sclerotized, associated C and MA, distal portion branched with hook-like apophysis (sub conductor), folded to hold embolus tip. MA distal pit accommodates BC lock mechanism, margin well sclerotized, unbranched, placed on tegulum, loop of sperm duct not clearly visible. TTA membranous, not sclerotized, tube-like elongated, retro laterally originated, very closely associated with C and E, apex with a knob in which embolus tip rests. E retro ventrally originated on tegulum, not hidden by cymbium, slightly curved, spiral tip entire not forked and needle-like with a median groove.

Female: unknown.

**Distribution.** See Fig 11.

### *Rhomphaea martini* sp. nov.

urn:lsid:zoobank.org:act:DA2EFE71-6A5E-4548-8A47-809919E6BADC

Figs 4P–4S, 5E, 5F, 6K, 6L, 7C and 7D

**Type material. Holotype:** ♂ (IFS_THE_757), Sri Lanka, Central Province, Nuwara Eliya District, Sita Eliya FR, 06°39’10’’N, 80°41’31’’E, 1743m, 20–22 November 2019, hand collection, leg S. P. Benjamin *et al*.

**Other material examined. Sri Lanka: *Sabaragamuwa Province*:** 1♀ (IFS_THE_50), Rathnapura District, Gilimale FR, 06°45’55.8’’N, 80°25’45.5’’E, 110m, 27 February 2007, Hand collection, S. P. Benjamin & Z. Jaleel. ***North western Province*:** ♀ (IFS_THE_25), Kurunegala District, Kurunegala, Nikaravatiya, March 2008, Hand collection, Z. Jaleel.

**Etymology.** The species is named after Sri Lankan novelists Lama Hewage Don Martin Wickramasinghe. He is known for his trilogy Gamperaliya, Yuganthaya, and Kaliyugaya. He also lived on the same street in Colombo as the second author.

**Diagnosis.** Males of this species differ from others congeners by the ocular area of male (projected anteriorly with a characteristic seta). Median eyes distantly separated from each other. Prosoma bordered laterally with two black dusty stripes continued from clypeus, stripes much darker and wider than those of *R*. *jacko* sp. nov. Opisthosoma of males shorter, triangular, rounded tip. Tibia of male palp much shorter than others. Embolus bifurcated with an apophysis. Females differ from others congeners by the conical, long, triangular opisthosoma with silvery patches and pointed tip, when compared to *R*. *shanthi* sp. nov. ventral epigynum socket (scape) smaller. CD with two loops, enters S anterioly (Fig 7C).

**Description.** Male (holotype): based on alcohol preserved specimens. Prosoma yellow, with a deep incision, two prominent lateral black bands extending from clypeus to chelicera, prosoma length 1. 25, prosoma width 0.73. Slanted clypeus, length 0.24. Sternum leaf shaped, longer than wide, widest at 1^st^ coxae and pointed at 4^th^ coxae, similar to prosoma in coloration and pattern, two bands bordering lateral sides. Labium fused with sternum. Eye region elevated, projected anteriorly with a characteristic seta between AMEs, medians slightly larger than others, measurements: AME 0.1, ALE 0.1, PME 0.1, PLE 0.1, PME-PME 0.1, PLE-PLE 0.4, ALE-PME 0.1, ALE-PLE 0. Opisthosoma yellow, fully covered with bright round silver spots, dorsal line marked with some black patches, anterior opisthosoma wider than posterior, posterior opisthosoma bent dorsally and conically tapered towards posterior rounded tip, spinnerets situated angularly in the middle of opisthosoma, angle pedicel–spinnerets–opisthosoma tip approximately around 80°–90°, total length: pedicel–spinnerets 1.25, spinnerets–tip of posterior opisthosoma 1.75, opisthosoma width 0.87. Legsyellow, black–brown bands, longer. Leg measurements: leg I: 9.7 (3.65, 0.3, 2.75, 2.0, 1.0, leg II: 6.57 (2.0, 0.3, 0.8, 2.72, 0.75, leg III: 3.15 (Fm 1.1, Pt 0.25, Tb 0.7, Mt 0.65, Ta 0.45, leg IV: 5.4 (1.9, 0.3, 1.35, 1.25, 0.6). Palpyellowish-brown, measurements: Ti 0.38, cymbial longitudinal length 0.38. Tibia distal end slightly wider than base, base of tibia narrower than patella, tibial rim uniform with row of 2–3 long setae, 3–5 retro lateral trichobothria, 1 prolateral trichobothria. Cy entire, retro margin distal apophysis forms Chd. Chk narrow, facing downward with blunt tip. ST retro lateral margin entire not lobed. T less than half of cymbial cavity. C outgrown and fused to T, long tube-like, membranous, distal portion tapered, sclerotized needle-like tip parallel to embolus spiral-tip. MA membranous, not fused, mesial origin, unbranched, distal tip with a narrow pit for Chk. TTA located in between E and MA, unbranched, tapered apex, distal tip bent, forms needle-like hook, loosely associated E. E moderately elongated, bifid, narrow and cylindrical spiral tip, ventrally located on tegulum and not hidden by cymbium, lobbed embolic base.

**Female.** As in male, except these followings: Prosoma similar to opisthosoma in colour, thoracic region slightly concaved. Eye measurements: AME 0.1, ALE 0.1, PME 0.1, PLE 0.1, PME–PME 0.1, PLE–PLE 0.4, ALE–PME 0.1, ALE–PLE 0. Opisthosoma pale yellow with shiny silver spots and black-brown flecks, elevated, conical, extended beyond spinnerets, posterior section 4 times longer than anterior section and tapered, posterior end pointed with a characteristic seta. Angle pedicel–spinnerets–opisthosoma tip 130°–160°. Spinnerets similar to opisthosoma in colour, angularly located at 1\4^th^ part of opisthosoma. Leg measurements: leg I: 3.93 (1.56, 0.3, 0.81, 0.81, 0.45), leg II: 9.8 (4.1, 0.3, 2.9, 1.7, 0.8), leg III: 7.4 (3.2, 0.3, 1.9, 1.5, 0.5), leg IV: 5.6 (2.1, 0.3, 1.6, 1.1, 0.5). Epigynum as in (Fig 7D), ventral irregular. CD with two loops, enters S anterioly (Fig 7C).

**Distribution.** See Fig 11.

### *Rhomphaea marani* sp. nov.

Figs 4J–4L, 4M-4O, 5G, 5H, 6M–6P, 7E and 7F

urn:lsid:zoobank.org:act:022A514B-F850-4A7A-92F2-6907A2DDACEA

**Type material. Holotype:** ♂ (IFS_THE_292), Sri Lanka, Sabaragamuwa Province, Rathnapura District, Sinharaja FR, Kudawa, secondary forest, 06°26’1’’N, 80°25’7’’E, 442m, 5 March 2019, beating, leg S. P. Benjamin *et*.*al*.

**Other material examined: Sri Lanka: *Uva Province*:** 1♂ (IFS_THE_24), Monaragala District, Nilgala FR (near Makara), 07°11’08.79’’N, 81°24’24.18’’E, 122m, 12 July 2017, beating, N. P. Athukorala & I. S. Illeperuma; ***Southern Province*:** 1♂ (IFS_THE_789), Galle District, Kanneliya FR, 06°15’04’’N, 80°20’18’’E, 30m, 18 February 2020, hand collection, leg S. P. Benjamin *et al*; 1♀,1♂ (IFS_THE_806–807): same locality and collection data as above; ***Sabaragamuwa Province*:** 1♀ (IFS_THE_047), Rathnapura District, Gilimale FR, 06°45’55.8’’N, 80°25’45.5’’E, hand collection, 27 February 2007, leg S. P. Benjamin & Z. Jaleel.

**Etymology.** This species is named after the author’s brother Seermaran for assistance and support during this study.

**Diagnosis.** This species differs from others congeners by the following characters: prosoma shaded with brown with a median yellow stripe. Thoracic region with deep incision. Eye region not projected as *R*. *martini* sp. nov. furrowed between median eyes. Narrow cylindrical opisthosoma with dark reddish-brown patches all over the body, which gives them a unique twig-like appearance. Male palp with elongated tibia and bifid embolus. Epigynum with large S, short CD. CD longer in both *R*. *shanthi* sp. nov and *R*. *martini* sp. nov.

**Description.** Male (holotype): based on alcohol preserved specimens. Prosoma pale yellow with two broad black bands on both sides extended up to clypeus, deep incision in the middle, two humps, prosoma length 1.05, prosoma width 0.75. Clypeus length 0.27. Sternum pale yellow with similar pattern of patches, wider than long, widest at 1^st^coxae and pointed at 4^th^coxae. Eye region projected anteriorly with usual eye band arrangement, larger AMEs, medians separated from each other and close to laterals, measurements: AME 0.1, ALE 0.1, PME 0.1, PLE 0.1, PME–PME 0.1, PLE–PLE 0.3, ALE–PME 0.1, ALE–PLE 0. Opisthosoma narrow cylindrical, pale yellow with prominent silver and brownish black patches, similar pattern of patches on ventral side, same width throughout the whole length, posterior region almost two times longer than anterior, posterior end shaded with dark brown and with 3–4 characteristic small humps, cross section of opisthosoma end triangular, length Pedicel–spinnerets 0.8, spinnerets–tip of opisthosoma 1.9, opisthosoma width 0.66. Spinnerets located in 1/3^rd^ length of opisthosoma anteriorly and positioned angularly. Legs; pale yellow, with black bands. Palp similar to legs in coloration and pattern of bands, measurements: length of palp tibia 0.7, length of cymbium 0.4. Tibia distal end slightly wider than base and tapered, base narrower than patella at its articulation, tibia rim exaggerated in the middle and facing bulb, scoop shaped, rim with regularly arranged row of uniform strong long setae, 4–5 retro lateral trichobothria, 1–2 pro lateral trichobothria. Cymbiumentire without cymbial hood. Chk distally placed inside of the cymbium, facing downward, distal portion tapered to a sharp tip. MA with distal narrow pit where Chk locks. ST large. T not larger than half of the cymbial cavity, with tegular pit at its ectal margin connected to embolus base. Conductor extension of tegulum, located between embolus and MA, membranous, larger and broader, distal portion bent but not folded. MA mesial originated, outgrowth of tegulum, unbranched, loop of sperm ducts not clearly visible, narrow pit-like hood on the distal MA. TTA membranous, long tube-like, apical knob of TTA overlaid on embolus spiral tip, closely associated with C and E, attached to T via extra tegular apophysis (ETA). ETAlocated in the ectal margin of tegulum and closely related to TTA. Bifid embolus with embolus apophysis, embolus base lobed and placed retro ventrally. Embolus spiral narrow, cylindrical and tapered distally to form a needle-like sharp tip.

**Female.** All characters as in male, except the followings: prosoma almost whole area black shaded, yellow star-like mark in the thoracic region, wider than long, median incision which leads to hump formation, back hump slightly elevated at the same level of height of eye projection, measurements: prosoma width 0.6, length 0.875. Clypeus yellow, black shaded, slightly slanted. Measurements: AME 0.1, ALE 0.06, PME 0.06, PLE 0.06, PME–PME 0.1, PLE–PLE 0.3, ALE–PME 0.1, ALE–PLE 0. Opisthosoma yellow with reddish brown and silver patches, patterns and colorations similar to male but darker, bigger size, shorter and wider compare to male, ’V’ shaped, cylindrical, posterior opisthosoma 1.5 times longer than anterior part, posterior opisthosoma tip has 4 small humps, total length: pedicel–spinnerets 1.0, spinnerets–tip of opisthosoma1.37, opisthosoma width 0.84. Epigynum as in (Fig 7F), with prominent scape, CD simple, straight, short, entering spermathecae basally (Fig 7E).

**Distribution.** See Fig 11.

*Genus Neospintharus Exline*, *1950*. *Neospintharus* Exline, 1950: 112. Type species: *Argyrodes parvus* Exline & Levi, 1962.

**Diagnosis:** Prosoma of male with anteriorly projected caput process and clypeus separated by a groove, two horn-like projections finished with long setae. Anterior median eyes in males placed on both sides of groove between eye projection and clypeal processes. Labium fused with sternum and not re-bordered. Opisthosoma triangular, posterior opisthosoma tip bifurcated, accommodates humps or forked (fish tail-like) process. Conductor distal tip is entire and lobed. TTA with enlarged and ridged distal tip. Embolus short or medium size.

**Composition.** *N*. *baboquivari* (Exline & Levi, 1962), *N*. *baekamensis* Seo, 2010, *N*. *bicornis* (O. Pickard-Cambridge, 1880), *N*. *concisus* (Exline & Levi, 1962), *N*. *fur* (Bösenberg & Strand, 1906), *N*. *furcatus* (O. Pickard-Cambridge, 1894), *N*. *nipponicus* (Kumada, 1990), *N*. *obscurus* (Keyserling, 1884), *N*. *parvus* Exline, 1950, *N*. *rioensis* (Exline & Levi, 1962), *N*. *syriacus* (O. Pickard-Cambridge, 1872), *N*. *triangularis* (Taczanowski, 1873), *N*. *trigonum* (Hentz, 1850).

**Distribution.** Argentina, Brazil, Canada, Caribbean, China, Japan, Korea, Mexico, Peru, Panama to Ecuador, USA.

### Key to the males of *Rhomphaea* and *Neospintharus* of Sri Lanka

**Fig 8:**
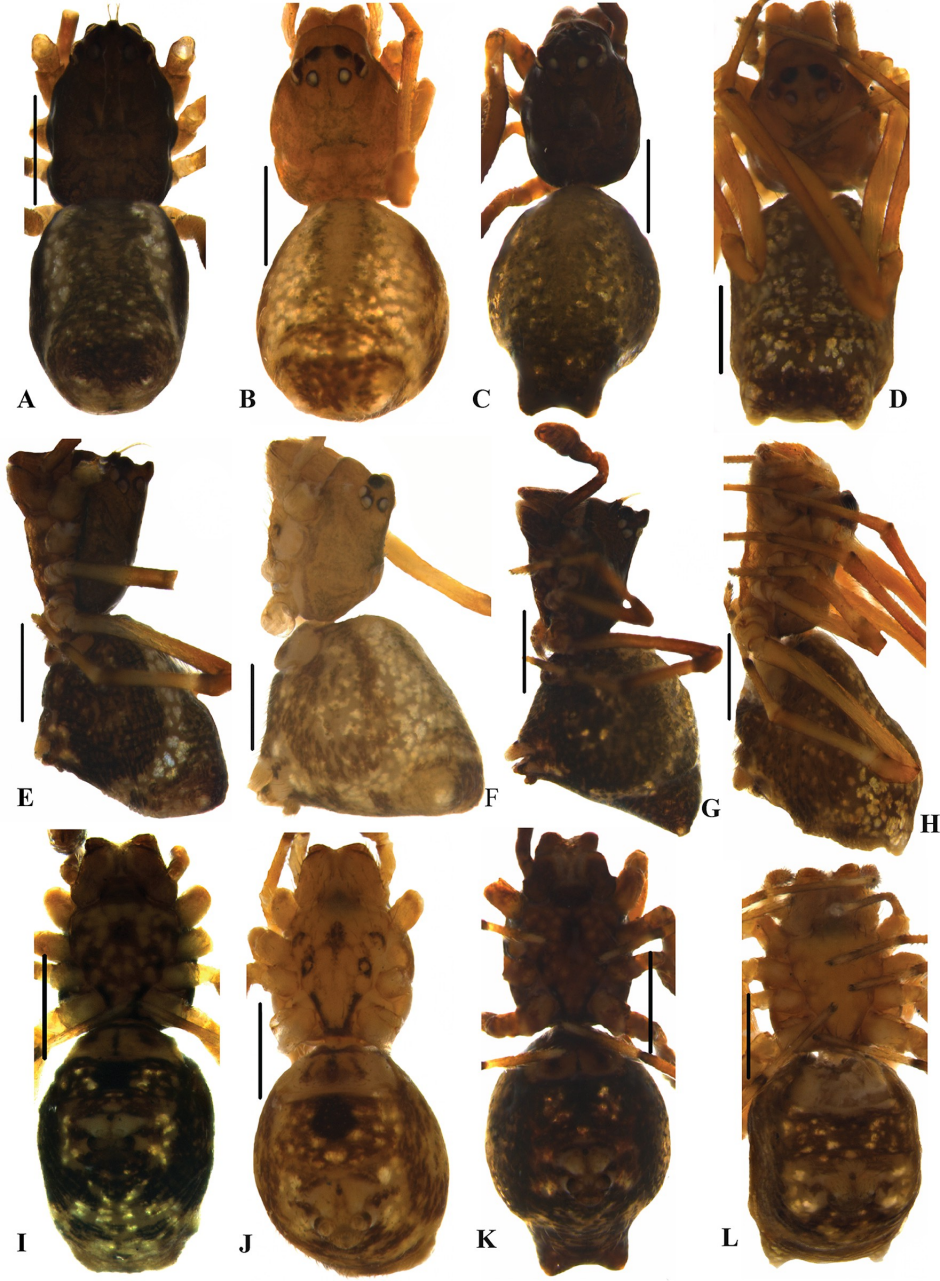
Habitus of *Neospintharus* species of Sri Lanka. A–B, E–F, I–J *N*. *kandelensis* sp. nov. A, C, E, G, I, K male habitus; B, D, F, H, J, L female habitus. C–D, G–H, K–L *N*. *ohiyiaensis* sp. nov. A–D dorsal view; E–H lateral view; I–L ventral view. Scale bars = 0.5 mm (A–L).

1. Species with elongated; cylindrical or boomerang shaped abdomen (Fig 4A–4C, 4G–4I, 4J–4L and 4P–4R) 2
  Species with short; triangular shaped abdomen (Fig 8) 3
2. Embolus bifurcated (Fig 6K and 6M) 4
  Embolus not bifurcated (Fig 6A and 6F) 5
3. E tapers from the base upwards, head and clypeal processes slender (Figs 8E and 10A) *N*. *kandelensis* sp. nov.
  E tapers from the midpoint upwards, head and clypeal processes are not slender (Figs 8C and 10B) *N*. *ohiyiaensis* sp. nov.
4. Tibia long, rim scoop shaped (Fig 6M) *R*. *marani* sp. nov.
  Tibia short, rim flat (Fig 6K) *R*. *martini* sp. nov.
5. Tibia rim scoop shaped, TTA apex not forked (Fig 6F) *R*. *jacko* sp. nov.
  Tibia rim not scoop shaped, TTA apex forked (Fig 6A) *R*. *shanthi* sp. nov.

### Key to the females of *Rhomphaea* and *Neospintharus* of Sri Lanka

(females of *R*. *jacko* sp. nov. unknown)

1. Species with elongated and/or cylindrical abdomen (Fig 4D–4F, 4M–4O and 4S) 2
  Species with short; triangular shaped abdomen (Fig 8B, 8D, 8F, 8H, 8J and 8L) 3
2. CD very long (Fig 7E and 7F) 4
  CD short (Fig 7A–7D) *R*. *marani* sp. nov.
3. Smooth posterior opisthosomal border, CD longer (Figs 8A, 8B and 10D) *N*. *kandelensis* sp. nov.
  Curved posterior opisthosomal border, CD shorter (Figs 8C, 8D and 10F) *N*. *ohiyiaensis* sp. nov.
4. CD with one loop (Fig 7A) *R*. *shanthi* sp. nov.
  CD with two loops (Fig 7C) *R*. *martini* sp. nov.

### *Neospintharus kandelensis* sp. nov.

urn:lsid:zoobank.org:act:95846666-9185-4E48-A217-6531B1F29FD5

**Fig 9:**
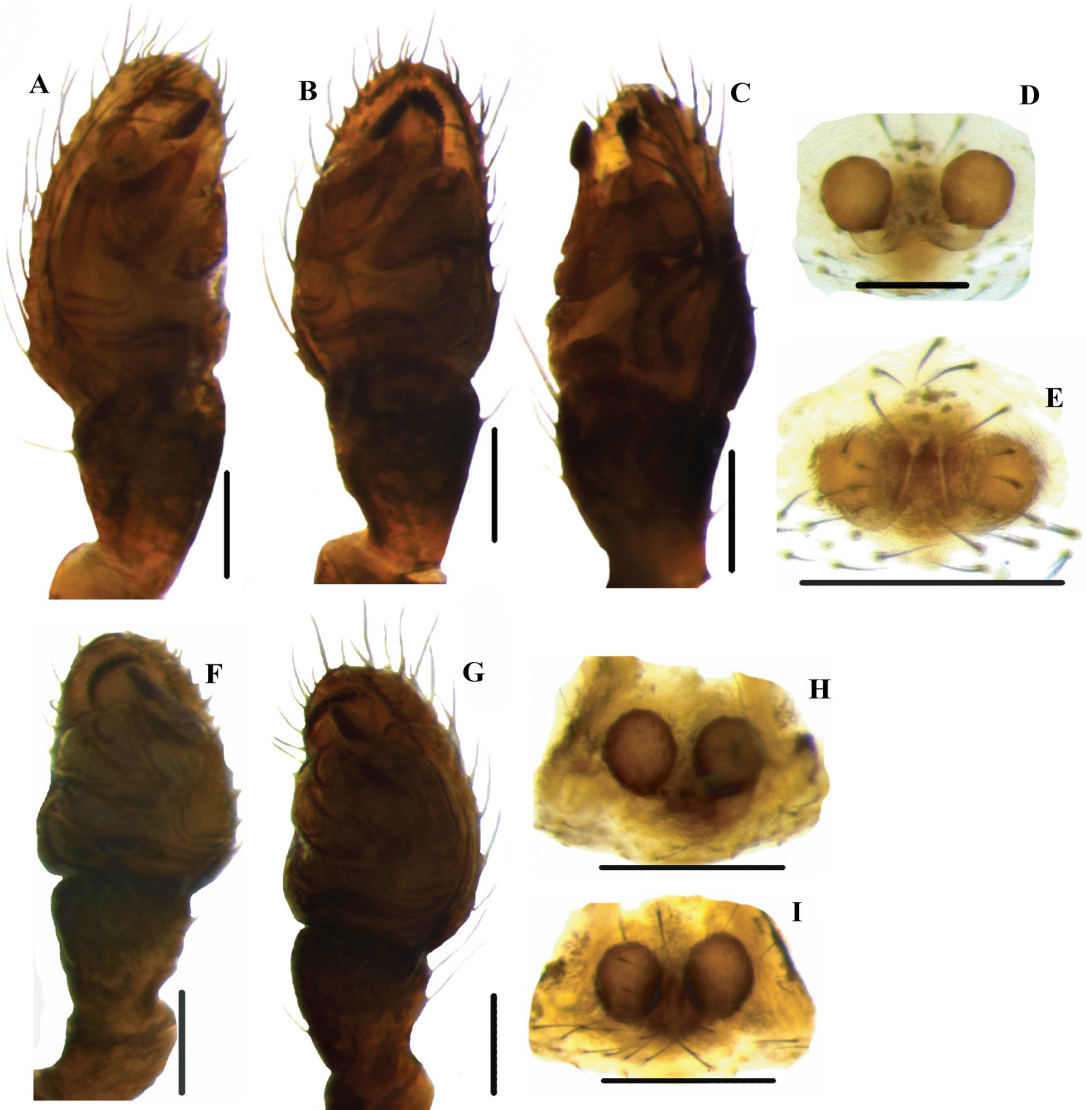
Genital morphology of *Neospintharus* species in Sri Lanka. A–E, *N*. *kandelensis* sp. nov. A–C, male palp (left). **A** prolateral view; **B** ventral view; **C** retrolateral view. D–E, Epigynum. D, dorsal view; E, ventral view. F–I, *N*. *ohiyiaensis* sp. nov. F, male palp (left), ventral view; G, same, retrolateral view; H, epigynum dorsal view; I, same, ventral view. Scale bars = 0.1 mm (A–D, F–G), 0.2 mm (E, H, I).

**Fig 10:**
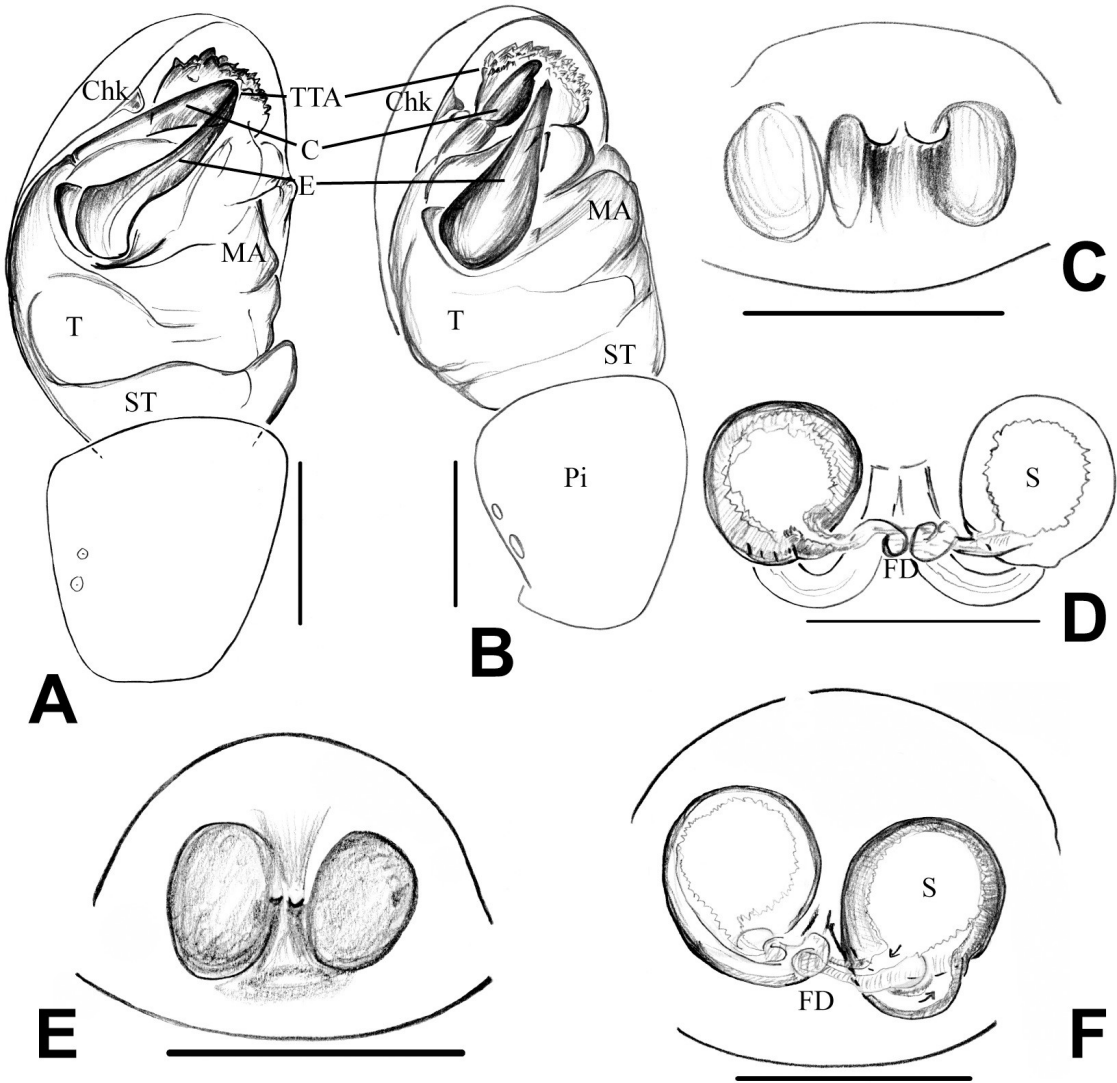
Genital morphology of *Neospintharus* species in Sri Lanka. A, male palp of *N*. *kandelensis*, ventral view; B, male palp of *N*. *ohiyiaensis*, ventral view. C, D *N*. *kandelensis*, epigynum, C dorsal view; D ventral view. E–F *N*. *ohiyiaensis*, epigynum, E dorsal view; F ventral view. Abbreviations = C, conductor; CD, copulatory duct; Chd, cymbial hood; Chk, cymbial hook; E, embolus; FD, fertilization duct; MA, median apophysis; S, spermathecae; ST, sub tegulum; T, tegulum; Ti, tibia; TTA, theridiid tegular apophysis. Scale bars = 0.1 mm (A–F).

Figs 8A, 8B, 8E, 8F, 8I, 8J, 9A–9E, 10A, 10C and 10D

**Type material. Holotype:** ♂ (IFS_THE_749), Sri Lanka, Central Province, Nuwara Eliya District, Kande Ela, 6.9° 13.3’34.56 N, 80.7°49.7’50.87’’ E, 1895m, hand collection, 22 November 2019, S. P. Benjamin *et al*. **Paratype:** ♀ (IFS_THE_748): Same locality and collection data as in holotype.

**Other material examined. Sri Lanka: *Central Province*:** ♂ (IFS_THE_744), Nuwara Eliya District, Kande Ela, 6.9°13.3’34.56’’N, 80.7°49.7’50.87’’E, 1895m, Hand collection, 22 November 2019, S. P. Benjamin *et al*; 2♀ (IFS_THE_755) (IFS_THE_760), Nuwara Eliya District, Sita Eliya forest reserve, 06°39’10’’N, 80°51’31’’E, 1743m, Hand collection, 22 November 2019, S. P. Benjamin *et al*; 1♀ (IFS_THE_815), Nuwara Eliya District, Horton Plains National Park, 06° 47’54’’N, 80°48’51’’E, 2000m, Beating, 21 February 2007, S. P. Benjamin *et al*; 1♂ (IFS_THE_763), Kandy, Deltota, Loolcondera Estate, 07°08’45’’N, 80°41’53’’E, 1480m, Beating, 15 November 2017, N. P Athukorala *et al*.

**Etymology.** This species is named after its type locality.

**Diagnosis.** *N*. *kandelensis* sp. nov. differs from *N*. *ohiyiaensis* sp. nov. by the smooth posterior opisthosomal border (Fig 8A and 8B). Further, males of *N*. *kandelensis* sp. nov. differ from *N*. *ohiyiaeinsis* sp. nov. by the short caput process as well as the clypeal process and E that tapers from the base upwards (Fig 10A). Females of *N*. *kandalensis* sp. nov. differ from *N*. *ohiyaenesis* sp. nov. by the comparatively longer CD (Fig 10D).

**Description.** Male (holotype): based on alcohol preserved specimens. Prosoma yellow-brown with some grey hue, wider, prosoma length 1.06, prosoma width 0.72. Caput process projected anteriorly to form a small high lobe with blunt tip of the process bearing 3–4 long and strong setae. Clypeal process slightly longer than head process, narrow, cylindrical, tapered towards tip bearing 3–4 long and strong setae as in caput process, projected parallelly to caput process, not equal length of caput process. Sternum, coxa pale yellow with some grey hue except coxa legs yellow-brown in colour. Sternum shield-like. Labium fused with sternum and not re-bordered. Eyes located between caput process and clypeal process, AMEs larger than PMEs, square orientation of AMEs and PMEs. Separation of posterior medians slightly more than the separation of anterior medians. Eye measurements: AME 0.1, ALE 0.06, PME 0.1, PLE 0.06, PME-PME 0.1, PLE-PLE 0.36, ALE-PME 0.06, and ALE-PLE 0 (fused laterals). Opisthosoma nearly triangular, light grey with silvery hue or spots, dorsal opisthosoma more concentrated with a band of silver hue, posterior opisthosoma blunt darker grey hue and characteristically both lateral sides with a larger circular marking filled with darkened grey flecks. Spinnerets; light grey, placed in the middle and lower edge of opisthosoma, total length 1.98, opisthosoma length 1.08 and opisthosoma width 0.74. Leg measurements: leg I: 4.48 (1.54, 0.32, 1.2, 0.78, 0.64), leg II: 3.3 (1.02, 0.28, 0.8, 0.6, 0.6), leg III: 1.64 (0.64, 0.22, 0.38, 0.3, 0.1), leg IV: 2.67 (1.025, 0.25, 0.5, 0.475, 0.425). Palp distinctively broadened, tibial distal end asymmetrically slightly protruding, tibial rim facing bulb of the palp. Tibia with two retrolateral trichobothria and one prolateral trichobothria. Cymbium entire retro margin, distal pro margin, no cymbial hood. Chk blunt tip placed distally and facing downward. T size less than half of cymbial cavity and located on top of ST. C originated from T, entire not folded or grooved and distal tip of embolus and conductor associated with each other, C distal portion not pointed, subequal to base, sclerotized. MA mesially originated, easily distinguishable sclerite, placed on tegulum. TTA unbranched, located between MA and C, closely associated with E and C, distal tip enlarged and strongly sclerotized ridged surface. E originated retro ventrally on tegulum, embolus spiral short, embolus base slightly lobbed, measurements: palp tibia 0.2 and length of cymbium 0.32.

**Female (paratype).** based on alcohol preserved specimens. All characters as in male, except the following: larger than males, total length 2.12, pattern similar to males, darker and browner than males with silvery spots. Prosoma; middle dark patches, posterior wider, length 1.04, width 0.74. Clypeus without modifications, high, rounded. Eye region; slightly elevated with a groove under AMEs, measurements: AME 0.1, ALE 0.1, PME 0.1, PLE 0.1, PME–PME 0.1, PLE–PLE 0.3, ALE–PME 0.3 and ALE–PLE 0 (fused laterals). Pedipalp and legs pale yellow. Opisthosoma length 1.3, opisthosoma width 1.0. Leg measurements: leg I: 4.44 (1.62, 0.27, 1.25, 0.75, 0.55), leg II: 3.41 (1.1, 0.27, 0.82, 0.62, 0.6), leg III: 2.16 (0.75, 0.22, 0.4, 0.37, 0.42), leg IV: 3.36 (1.2, 0.3, 0.72, 0.62, 0.52). Epigynum with a central sclerotized plate. Copulatory pore wide and anterior to plate. CD short, loop posterior to S. S rounded. FD, short hook-like.

**Variation:** Females variable in size: 1.83–2.20. Male and female Color varies from light brown to reddish brown.

**Fig 11:**
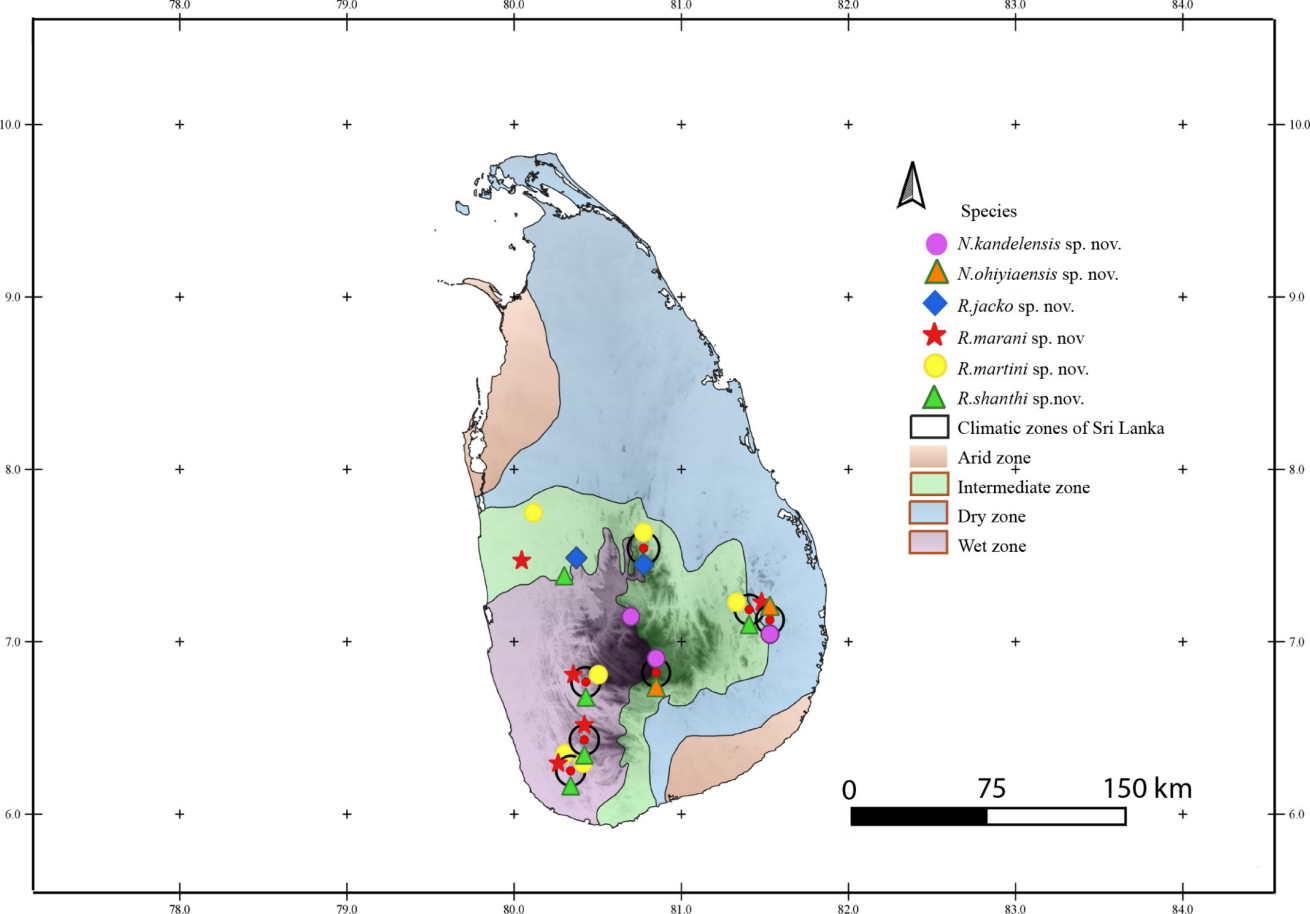
Distribution of *Rhomphaea* and *Neospintharus* species described from Sri Lanka. The localities of *R*. *shanthi* sp. nov. are represented with green triangles, *R*. *jacko* sp. nov. with blue diamond, *R*. *martini* sp. nov. with yellow circle, *R*. *marani* sp. nov. with red star, *N*. *kandelensis* sp. nov. with purple circle and *N*. *ohiyiaensis* sp. nov. with orange triangle outlined in green.

**Distribution.** See Fig 11.

### *Neospintharus ohiyiaensis* sp. nov

urn:lsid:zoobank.org:act:2E004C4A-3FB4-4726-BAA3-ED73039476DE

Figs 8C, 8D, 8G, 8H, 8K, 8L, 9F–9I, 10B, 10E and 10F

**Type material. Holotype:** ♂ (IFS_THE_57), Sri Lanka, Uva Province, Badulla District, Ohiya, 06°50’32’’N, 80°53’05’’E, 1370m, 30 August 2011, Athukorala *et al*. **Paratype:** ♀ (IFS_THE_58): Same locality and collection data as holotype.

**Other material examined. Sri Lanka: *Central Province*:** 2♀ (IFS_THE_819–820), Nuwara Eliya District, Kande-Ela, 6.9°13.3’34.56N, 80.7°54.7’50.87’’E, 1895m, Hand collection, 22 November 2019, S. P. Benjamin *et al*.

**Etymology.** This species is named after its type locality.

**Diagnosis:** Males and females of *N*. *ohiyiaensis* sp. nov. differ from *N*. *kandelensis* sp. nov. by their curved posterior opisthosomal border and longer clypeus (Fig 8C, 8D, 8K and 8L). Females differ by the shorter CD (Fig 10F).

**Description.** Male: (holotype): based on alcohol preserved specimens. Total length 2.12. Prosoma; blackish brown, wide and long, prosoma length 0.84, prosoma width 0.72. Clypeal process short, long, directed forward, parallel to caput process. Tip of caput process and clypeus process with 3–4 setae. Sternum with reddish brown hue. Ocular area, elevated, horn-like, slightly bent, tapered and projected. AMEs placed in between caput and clypeus process, larger than PMEs, fused laterals, Orientation median eyes in a square, measurements: AME 0.1, ALE 0.06, PME 0.08, PLE 0.06, PME-PME 0.12, PLE-PLE 0.32, ALE-PME 0.1 and ALE-PLE 0 (fused laterals). Opisthosoma; pale yellow, dusted with grey hue and silver spots, upper side of posterior opisthosoma prolonged, directed ventrally and tapered to form a silver bifid reddish-brown forked apex, width of fork tail 0.44, ventral side of anterior opisthosoma dusted with reddish brown and silver patches, lateral sides with reddish-brown circular markings, opisthosoma length 1.32, opisthosoma width 0.96. Legs long, slender, reddish brown. Spinnerets located middle of opisthosoma. Leg measurements: leg I: 3.91 (1.32, 0.32, 0.95, 0.75, 0.57), leg II: 3.14 (1.0, 0.3, 0.72, 0.6, 0.52), leg III: 1.84 (0.62, 0.2, 0.37, 0.3, 0.35), leg IV: 2.64 (0.9, 0.27, 0.62, 0.4, 0.45). Palp similar to legs in colouration, measurements: tibia 0.18, length of cymbium 0.3. Conductor tube-like elongated, slightly curved ventrally, tip slightly lobbed, sclerotized. Embolus moderate in size, narrowed, tip spiralled.

Female (**paratype**): based on alcohol preserved specimens. All characters as in male, except the followings: slightly bigger and darker than males. Prosoma yellow, wider than long, with a small grey hue, prosoma length 0.97, prosoma width 0.77. Sternum and labium pale yellow. Clypeus high and rounded. Eye region slightly elevated, measurements: AME 0.08, ALE 0.05, PME 0.1, PLE 0.05, PME-PME 0.05, PLE-PLE 0.12, ALE-PME 0.34, ALE-PLE 0 (fused laterals). Opisthosoma with silver, reddish-brown spots on lateral sides, big circular portion filled with reddish-brown spots, characteristic hump in posterior end of opisthosoma, width of hump range 0.7–0.74, forked tail facing downward, rusty reddish-brown hue. Total length 2.42, opisthosoma length 1.47, opisthosoma width 0.95. Legs and prosoma similar in colouration.Leg measurements: Leg I: 4.74 (1.62, 0.37, 1.35, 0.75, 0.65), leg II: 3.39 (1.2, 0.3, 0.77, 0.5, 0.625), leg III: 2.34 (0.77, 0.25, 0.45, 0.42, 0.45), leg IV: 3.29 (1.32, 0.3,0.72, 0.5,0.45). Epigynum well sclerotized, copulatory duct curved and loop relative to spermathecae base much closer.

**Variation:** Females vary considerably in opisthosoma colouration (brown to reddish brown), size and distribution of silver spots vary from smaller and more concentrated to larger and less concentrated and size (2.0–3.0).

**Distribution.** See Fig 11.

## Supporting information

S1 Fig Phylogeny of the subfamily Argyrodinae obtained from ML analysis of concatenated molecular data. (TIF)

S2 Fig Phylogeny of the subfamily Argyrodinae obtained from ML analysis of the *CO1* single gene molecular matrix. (TIF)

S3 Fig Phylogeny of the subfamily Argyrodinae obtained from ML analysis of the *16S* single gene molecular matrix. (TIF)

S4 Fig Phylogeny of the subfamily Argyroidinae obtained from ML analysis of the *28S* single gene molecular matrix. (TIF)

S5 Fig Phylogeny of the subfamily Argyrodinae obtained from Bayesian analysis of *CO1* single gene molecular matrix. (TIF)

S6 Fig Phylogeny of the subfamily Argyrodinae obtained from Bayesian analysis of *16S* single gene molecular matrix. (TIF)

S7 Fig Phylogeny of the subfamily Argyrodinae obtained from Bayesian analysis of *28S* single gene molecular matrix. (TIF)
