# Supplementary figures and images for: Origin and diversification of free-living stick spiders of Sri Lanka including the description of four new species of Rhomphaea L. Koch, 1872 and two new species of Neospintharus Exline, 1950
Source: PLoS One. 2022 Sep 7;17(9):e0273105. doi: 10.1371/journal.pone.0273105 (PMC9451084; doi:10.1371/journal.pone.0273105)

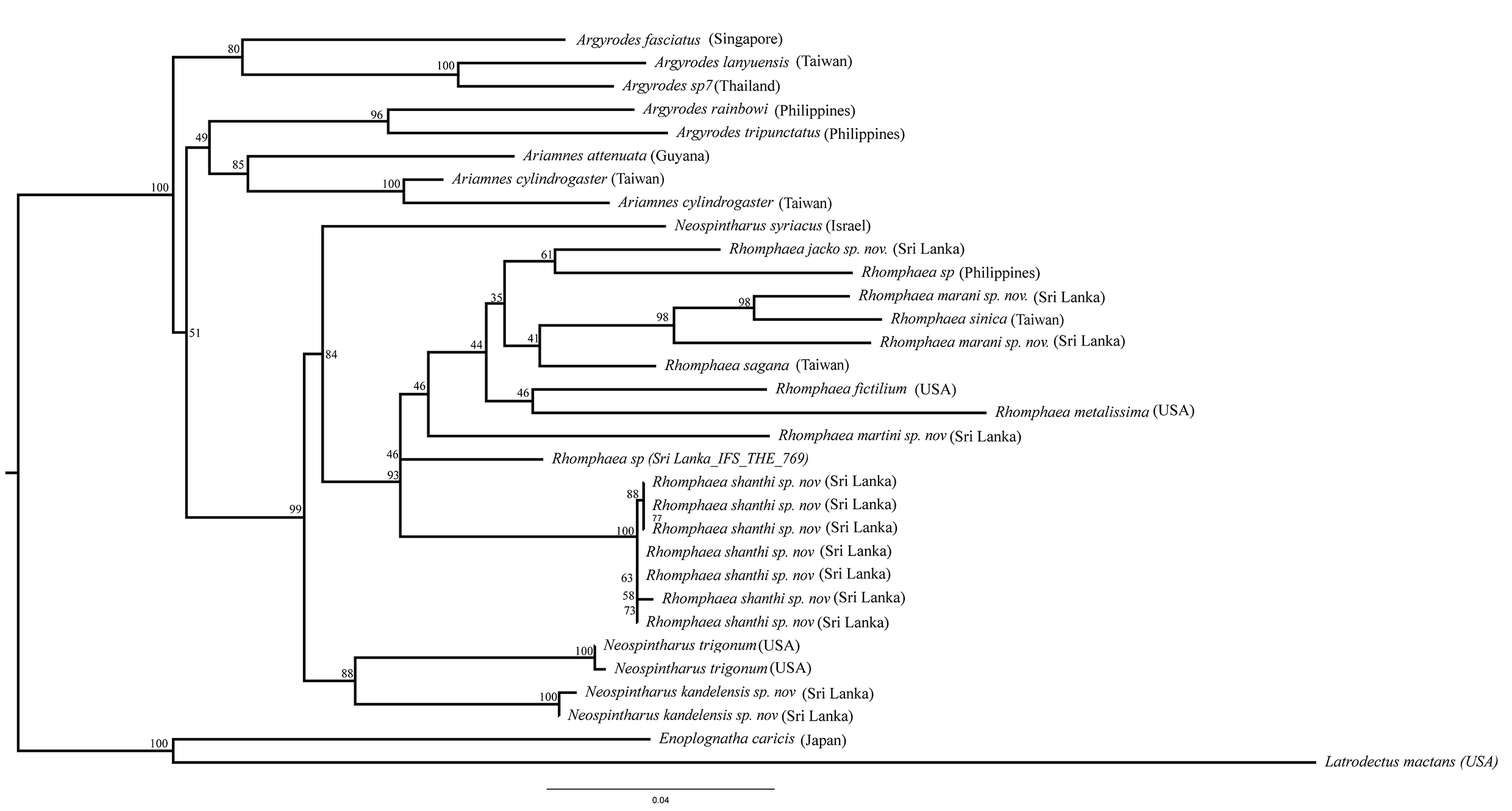

Supplement: S1 Fig — (TIF) [file pone.0273105.s001.tif]

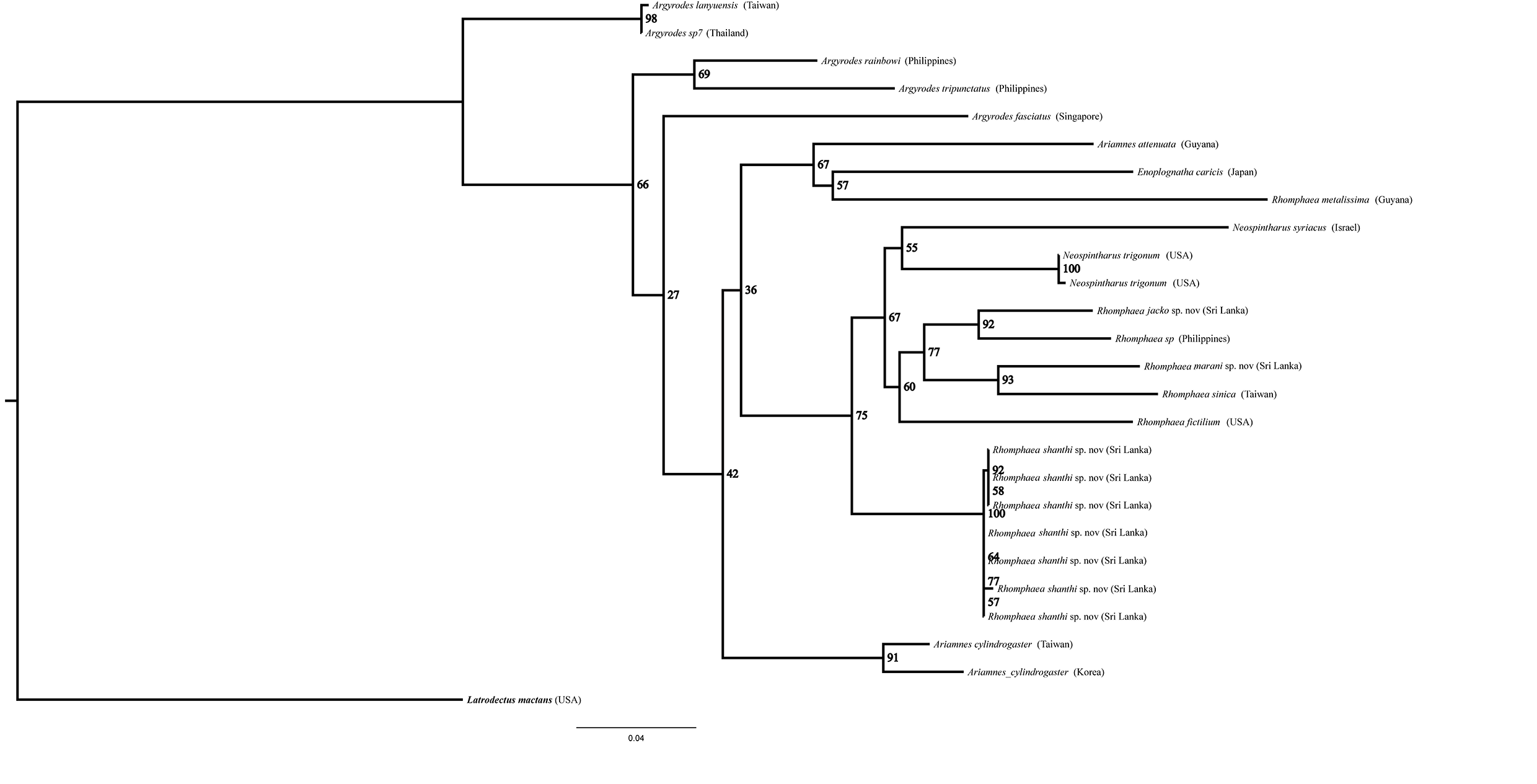

Supplement: S2 Fig — (TIF) [file pone.0273105.s002.tif]

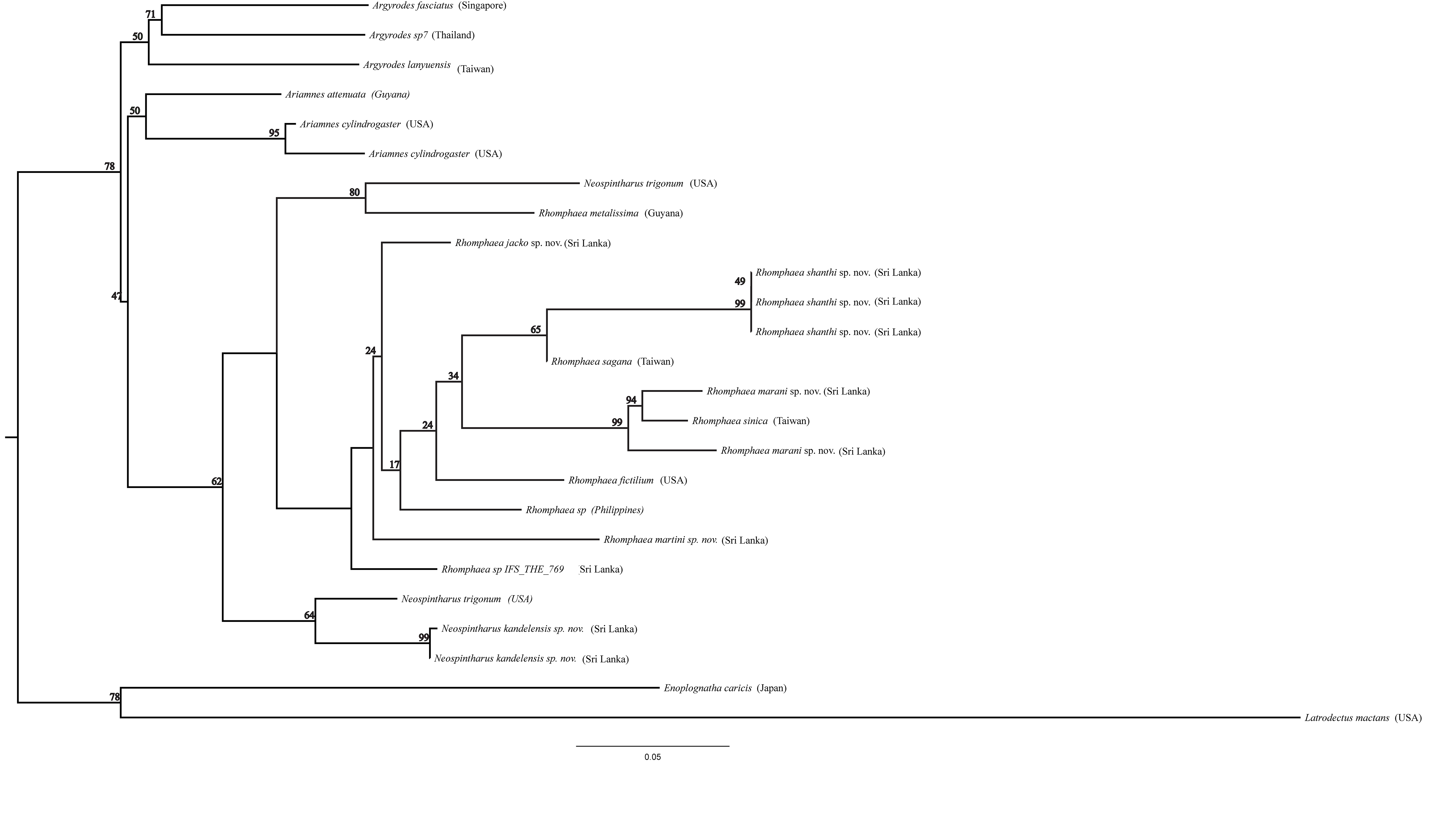

Supplement: S3 Fig — (TIF) [file pone.0273105.s003.tif]

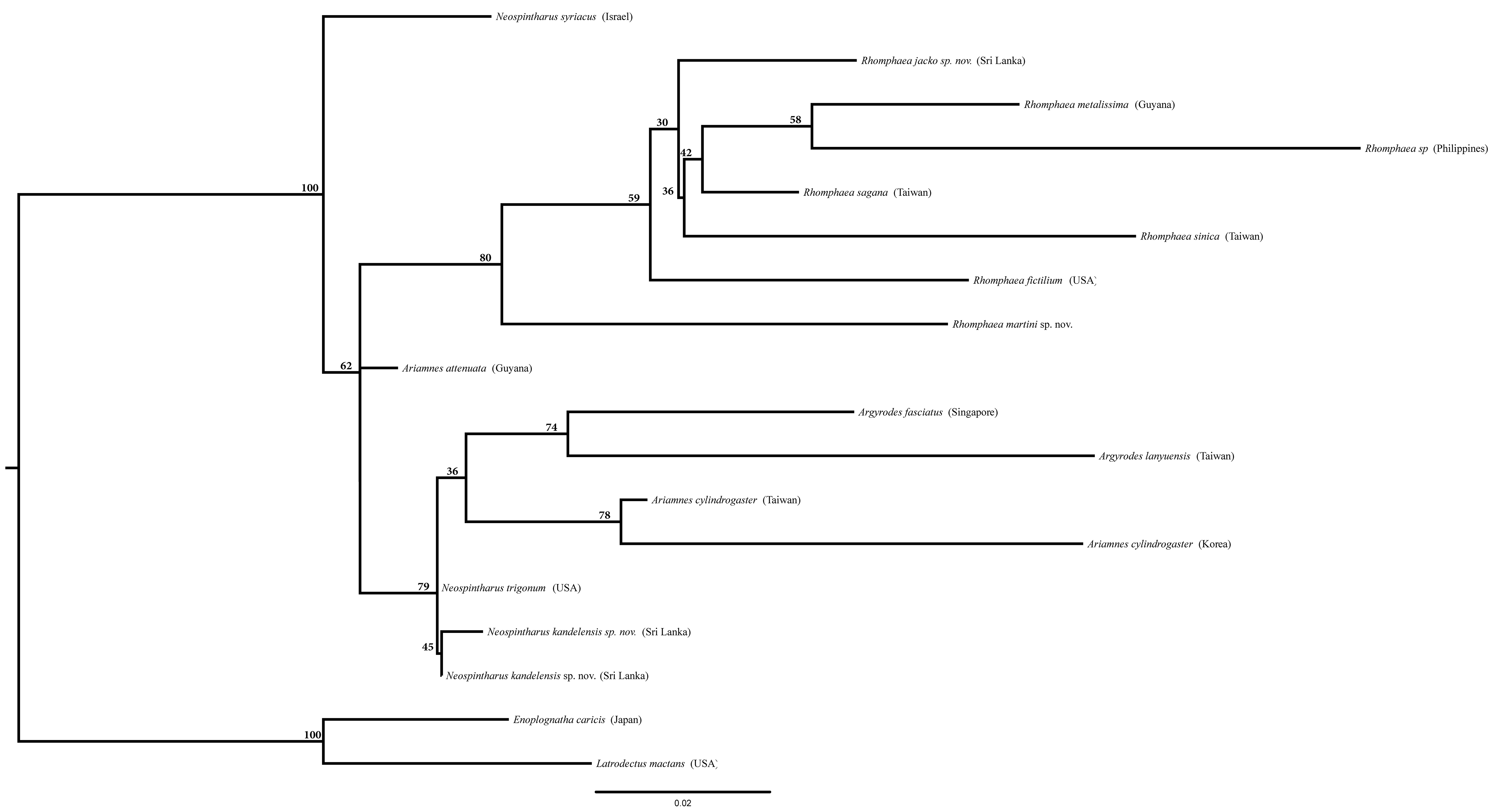

Supplement: S4 Fig — (TIF) [file pone.0273105.s004.tif]

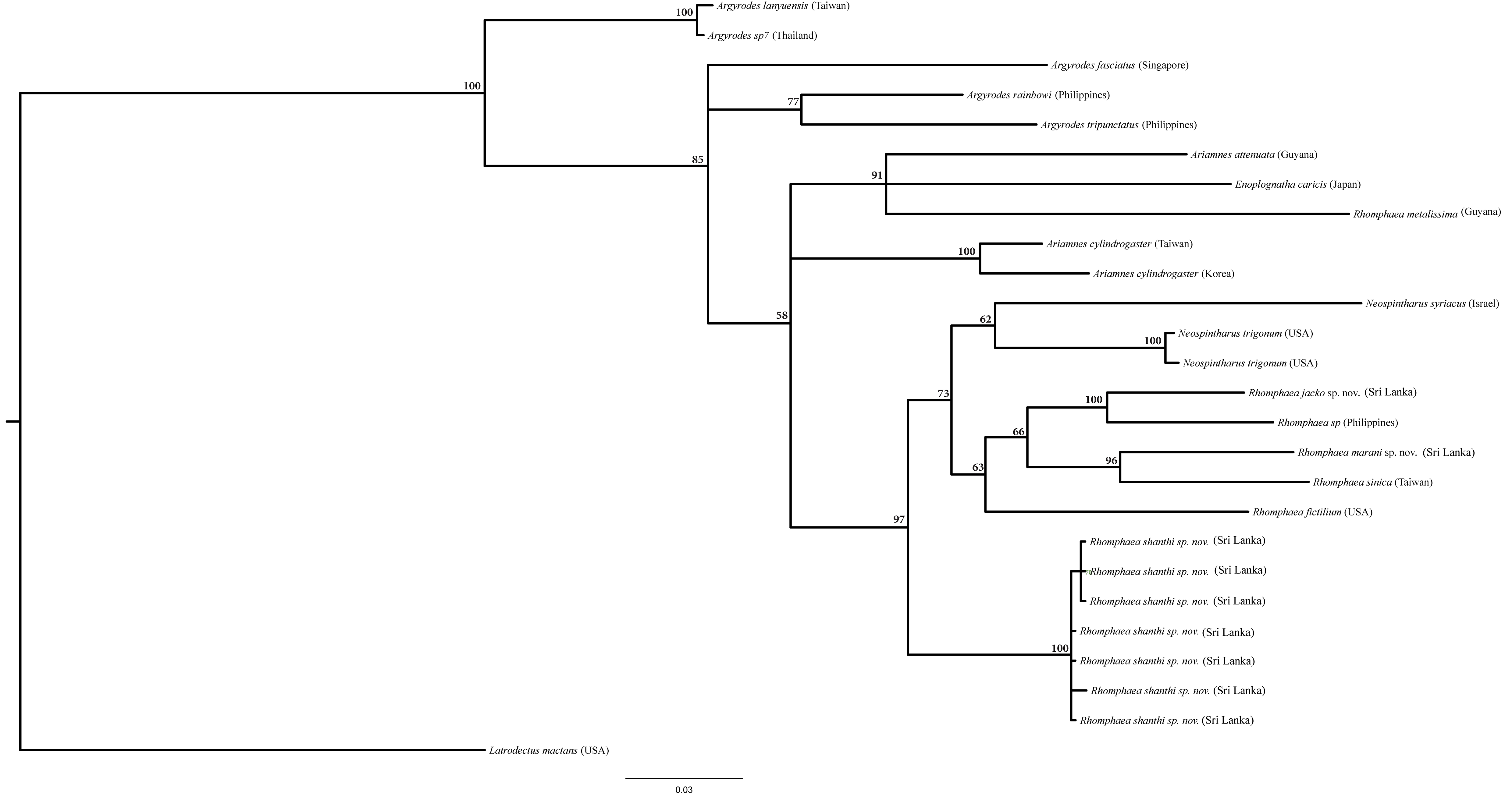

Supplement: S5 Fig — (TIF) [file pone.0273105.s005.tif]

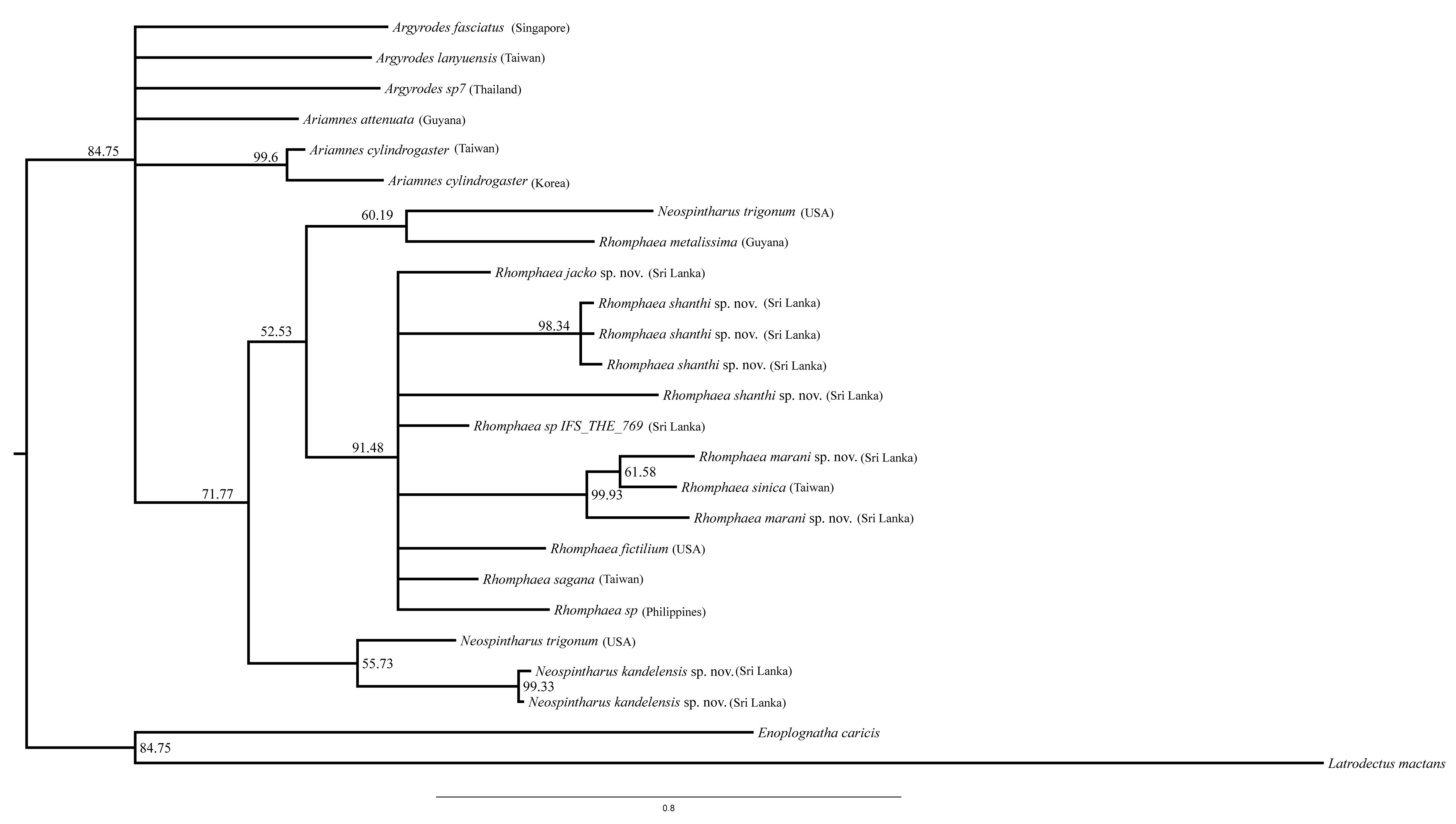

Supplement: S6 Fig — (TIF) [file pone.0273105.s006.tif]

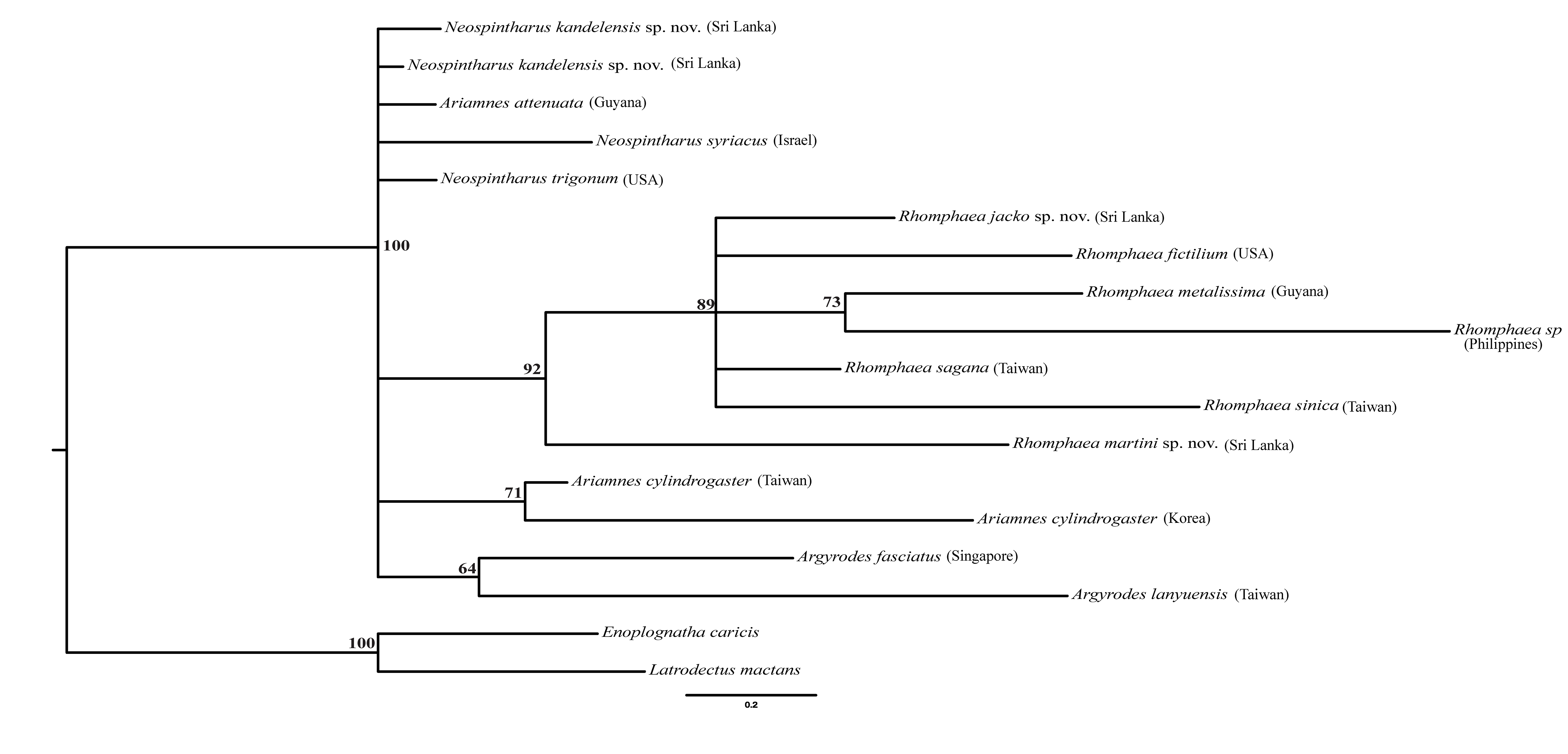

Supplement: S7 Fig — (TIF) [file pone.0273105.s007.tif]
